# Mapping epigenetic changes to the host cell genome induced by *Burkholderia pseudomallei* reveals pathogen-specific and pathogen-generic signatures of infection

**DOI:** 10.1038/srep30861

**Published:** 2016-08-03

**Authors:** Deniz Cizmeci, Emma L. Dempster, Olivia L. Champion, Sariqa Wagley, Ozgur E. Akman, Joann L. Prior, Orkun S. Soyer, Jonathan Mill, Richard W. Titball

**Affiliations:** 1 College of Engineering, Mathematics and Physical Sciences, University of Exeter, Exeter, United Kingdom; 2 University of Exeter Medical School, Exeter University, Exeter, United Kingdom; 3 College of Life and Environmental Sciences, University of Exeter, Exeter, United Kingdom; 4 School of Life Sciences, University of Warwick, United Kingdom; 5 Institute of Psychiatry, Psychology & Neuroscience, King’s College London, United Kingdom

## Abstract

The potential for epigenetic changes in host cells following microbial infection has been widely suggested, but few examples have been reported. We assessed genome-wide patterns of DNA methylation in human macrophage-like U937 cells following infection with *Burkholderia pseudomallei*, an intracellular bacterial pathogen and the causative agent of human melioidosis. Our analyses revealed significant changes in host cell DNA methylation, at multiple CpG sites in the host cell genome, following infection. Infection induced differentially methylated probes (iDMPs) showing the greatest changes in DNA methylation were found to be in the vicinity of genes involved in inflammatory responses, intracellular signalling, apoptosis and pathogen-induced signalling. A comparison of our data with reported methylome changes in cells infected with *M. tuberculosis* revealed commonality of differentially methylated genes, including genes involved in T cell responses (*BCL11B*, *FOXO1*, *KIF13B*, *PAWR*, *SOX4*, *SYK*), actin cytoskeleton organisation (*ACTR3*, *CDC42BPA*, *DTNBP1*, *FERMT2*, *PRKCZ*, *RAC1*), and cytokine production (*FOXP1*, *IRF8*, *MR1*). Overall our findings show that pathogenic-specific and pathogen-common changes in the methylome occur following infection.

Melioidosis is an infectious disease caused by the intracellular bacterial pathogen *Burkholderia pseudomallei*. A recent study has found that the incidence of this disease is likely to have been significantly underestimated^1^. *B. pseudomallei* adopts various strategies to survive and replicate within host cells^2^. Avoiding or resisting the antimicrobial activities in the phagosome of host cells^2^,^3^ allows escape into the cytosol, the induction of actin polymerization and cell to cell spreading^4^. The transcriptional response of humans or mice to *B. pseudomallei* infection reveals changes in the expression of multiple genes, including loci associated with the inflammatory and innate immune responses^5^,^6^,^7^. These changes might be driven actively by bacteria, or they may be the consequence of altered responses of host cells to the infection.

Recently, a role for epigenetic regulation of host cell function during bacterial infection has been suggested^8^,^9^. DNA methylation, occurring exclusively at cytosine residues in mammals, is one of the best understood epigenetic mechanisms, playing a key role in transcriptional regulation during development and being increasingly implicated in a range of non-infectious diseases^10^. Epigenetic processes can be dynamic: they are influenced upon exposure to a range of external environmental factors and stochastic events in the cell^11^.

Few studies have investigated epigenetic changes in response to infection but there are reports of altered patterns on methylation in cultured cells infected with *Helicobacter pylori*^12^,^13^, *Mycobacterium tuberculosis*^14^ or *Leishmania donovani*^15^ and evidence that in mice the gut flora influences methylation of the *IL-4* gene in intestinal epithelial cells resulting in the down-regulation of *TLR4* expression^16^. Some of these studies have focussed on selected genes^12^,^16^, others have profiled genome wide changes in the methylome^13^,^14^,^15^. These studies have revealed linkage between methylation events and the level of expression of associated gene(s).

In this study we determined genome-wide changes in DNA methylation in human macrophage-like U937 cells following infection by *B. pseudomallei*. We identify widespread changes in host cell DNA methylation following infection, and show that these are enriched in the vicinity of loci involved in inflammatory responses, intracellular signalling, apoptosis and pathogen-induced signalling. By comparing our data with the previously reported data we have identified genes, which show differential patterns of methylation in cells infected with different pathogens.

## Results

### Infection Model

**Figure 1:**
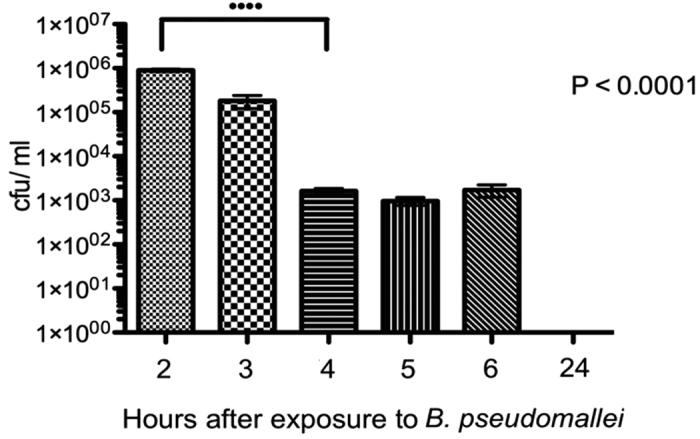
Bacterial loads at different time points in U937 cells infected with *B. pseudomallei* (MOI = 10). Bacterial load was measured as colony forming units (CFU).

U937 cells can mature and differentiate to adopt the characteristics of mature human macrophages^17^. We established the pattern of infection of U937 cells, which had been activated with interferon gamma (IFN-γ), by *B. pseudomallei* K96243 expressing red fluorescent protein (RFP). At 2 hours (T2) post infection, 74% of the human cells were infected (Supplementary Fig. 1) and the number of intracellular bacteria was 10^6^ CFU/ml (Fig. 1). After 4 (T4) or 6 hours (T6) post infection the number of intracellular bacteria had declined to 10^3^ CFU/ml. No intracellular bacteria were recovered at 24 hours post infection (T24). We and others^18^ have found that the activation of macrophages with IFN-γ dramatically enhances the ability of the cells to control infection with *B. pseudomallei* and this explains the progressive reduction in the number of intracellular bacteria.

### Multiple loci in U937 cells show reproducible changes in DNA methylation after B. pseudomallei infection

We infected U937 cells with *B. pseudomallei* and mapped changes in host cell DNA methylation at T2 and T4. DNA from the infected or uninfected (control) U937 cells was collected from two technical replicates and two experimental replicates at each time. We subsequently performed a second experiment following the same protocol but with additional sampling times included to provide samples at 1 hour (T1), 2 hours (T2), 3 hours (T3) and 4 hours (T4) post infection. The experimental design is shown in Supplementary Fig. 2.

DNA methylation was quantified using an Illumina 450K HumanMethylation array, with pre-processing, normalization and stringent quality control undertaken. In our first experiment we identified differentially-methylated positions (DMPs) between infected and uninfected cells at T2 or T4, allowing us to identify infection-induced DMPs (iDMPs).

**Figure 2:**
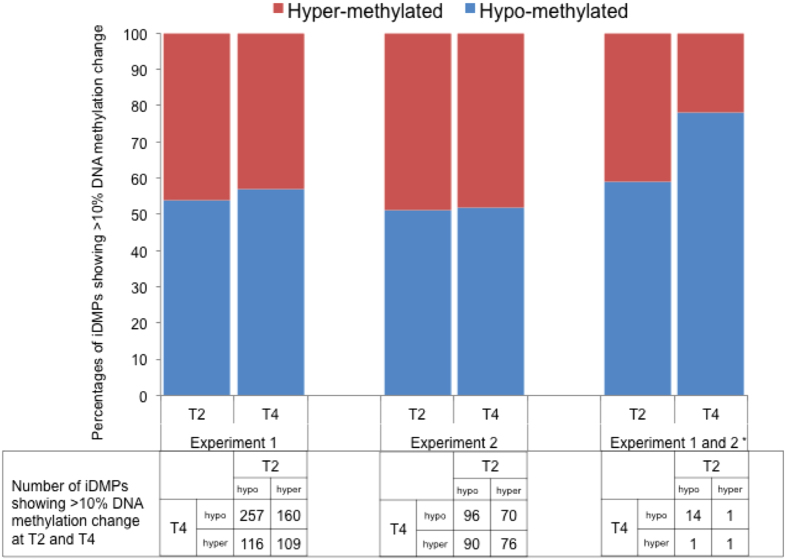
Quantification of Infection-induced iDMPs. *iDMPs conserved between the two experiments.

Reasoning that larger differences are potentially more biologically meaningful, our primary focus was on iDMPs characterized by >10% DNA methylation difference between groups^19^. We identified 10,279 iDMPs (54% hypo-methylated, 46% hyper-methylated) at T2 and 4850 iDMPs (57% hypo-methylated, 43% hyper-methylated) at T4, with 642 iDMPs differentially methylated at both T2 and T4 (Fig. 2). We next performed a second experiment to identify consistent changes and obtained a stringently-filtered dataset of 388 conserved iDMPs conserved between the two experiments and which were used for subsequent analysis. These DNA methylation changes were highly correlated across both experiments (T2: r = 0.83, p < 0.0001, Supplementary Fig. 3a; T4: r = 0.73, p < 0.0001, Supplementary Fig. 3b). 264 iDMPs were identical at T2, 141 iDMPs were conserved at T4 and 17 iDMPs conserved at both T2 and T4 (Supplementary Table 1).

The conserved iDMPs were not uniformly distributed across the genome (Supplementary Table 2). We found a highly-significant over- representation of infection-induced DNA methylation at probes located in the first exons of genes (compared to the frequency of positions in all probes on the Illumina 450K array analysed, relative enrichment (95% CI) = 3.34, P = 1.11 × 10^−22^).

**Figure 3:**
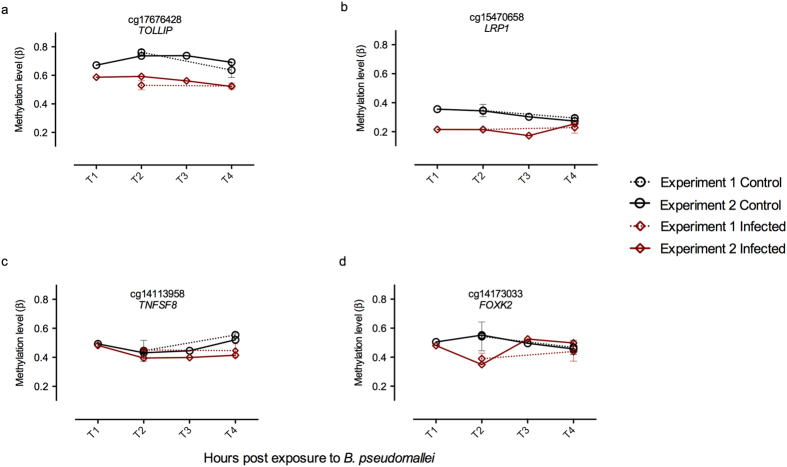
Differential methylation patterns. Each panel shows a representative gene, showing a particular temporal pattern as discussed in the main text and methods. The error bars represent the standard deviation of the methylation levels of the first experiment samples: (**a**) constant hypo-methylation; (**b**) early response hypo-methylation; (**c**) late response hypo-methylation; (**d**) transient response hypo-methylation.

In addition to quantifying DNA methylation at T2 and T4 in experiment 1 and 2, we profiled samples collected at T1 and T3 in experiment 2 (Supplementary Fig. 2). Distinct patterns of DNA methylation across the four time points were observed (Table 1); 21 probes (5.41%) showed consistent changes in DNA methylation across all time points in both experiments (for example cg17676428 (Fig. 3a)), 23 (5.93%) showed large changes at the early time-points that diminished at the later time-points (for example cg15470658 (Fig. 3b)), 55 (14.18%) displayed a lag in response, with DNA methylation changes only occurring at the later time points after infection (for example cg14113958 (Fig. 3c)), 99 (25.52%) showed a transient (for example cg14173033 (Fig. 3d)), and 190 (48.97%) an oscillatory response. The losses of methylation were more prominent compared to gains in DNA methylation patterns, especially in iDMPs exhibiting a constant response.

**Figure 4:**
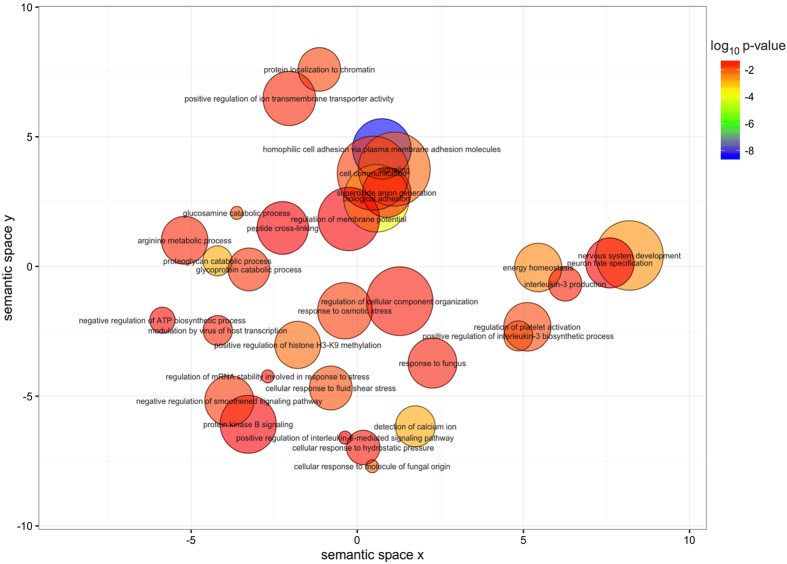
Gene ontology terms enriched (p < 0.05) in genes mapping to conserved iDMPs. The colour scale represents the p-values calculated using GOseq. The non-redundant gene ontology terms are clustered using REVIGO.

To explore the biological significance of the genes mapping to the set of iDMPs, we annotated their corresponding gene ontology (GO) term and searched for over-representation of categories using the package GOseq, which weights genes based on number of probes per gene. Enriched biological functions were summarised and visualised in Fig. 4 using semantic similarity, which provides a measure of functional similarity. Functionally similar GO terms appear closer in the plot.

### Comparison with publically available transcription data

We compared the iDMPs with transcriptomic data from a previous study of *B. pseudomallei* infection in humans which identified 2604 human genes, that were differentially expressed in patients with septicemic melioidosis^6^. Of these, 76 genes were annotated to the iDMPs we identified in our study. These included genes involved in immune system process (*BCL11B*, *CDKN1C*, *GLMN*, *HLX*, *IL1R2*, *IRF8*, *MAEA*, *MEF2C*, *MR1*, *NBEAL2*, *PRKCH*, *PTGDR2*, *STK3*, *TNFSF8*, *TRIM27*), response to stress (*ADRB2*, *APBB1IP*, *DTNBP1*, *FBXO31*, *MARCH1*, *MSRA*, *PKD2*, *SCARB1*, *ZMYND11*), and inflammatory response (*CD44*, *HDAC4*, *HIF1A*, *IL18RAP*, *TOLLIP*, *IER3*, *NT5E*).

### Comparison with publically available DNA methylation changes during infection with other pathogens

**Figure 5:**
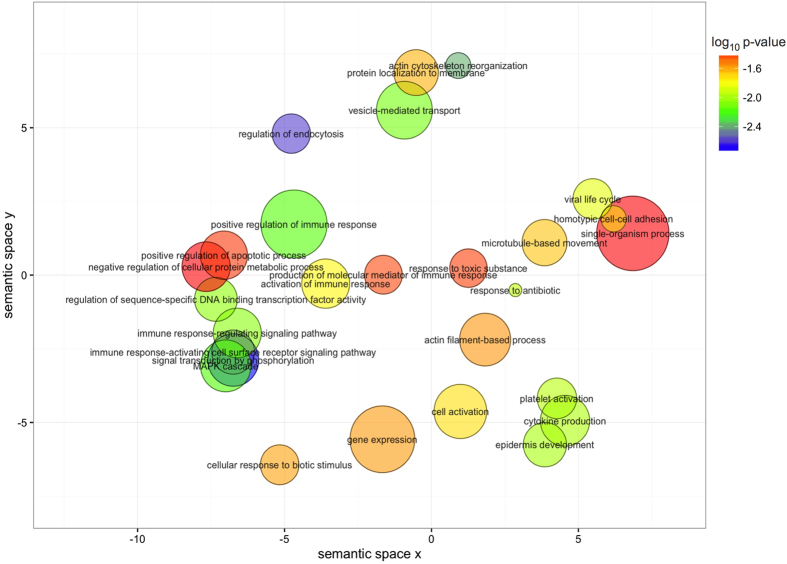
Gene ontology terms enriched (p < 0.05) in genes annotated to the iDMPs in *B. pseudomallei* and *M. tuberculosis* infections. The colour scale represents the p-values calculated using GOseq. The non-redundant gene ontology terms are clustered using REVIGO.

Innate immune cells, such as macrophages or dendritic cells, are recruited in response to pathogens to initiate defense mechanisms. While this study explores DNA methylation changes in macrophages, a recent study identified epigenetic regulation in human dendritic cells before and after *in vitro* infection (at 18 hours after infection) with *Mycobacterium tuberculosis*^14^. The proportions of iDMPs compared to the total number of sites probed were comparable to our study (0.000912 in our study; 0.000649 in the *M. tuberculosis* study). We did not identify any stringently-filtered iDMPs which were identical in cells infected with *B. pseudomallei* or with *M. tuberculosis* (Supplementary Fig. 4). However, when we considered the genes to which these iDMPs mapped we identified 121 genes (median distance of ~95 kb from the nearest transcription start site), which showed differential methylation in both studies (Supplementary Table 3). These included genes involved in T cell responses (*BCL11B*, *FOXO1*, *KIF13B*, *PAWR*, *SOX4*, *SYK*), actin cytoskeleton organization (*ACTR3*, *CDC42BPA*, *DTNBP1*, *FERMT2*, *PRKCZ*, *RAC1*), and cytokine production (*FOXP1*, *IRF8*, *MR1*) (Fig. 5).

## Discussion

This study expands the limited knowledge on the role of epigenetics during infection by reporting temporal changes to the genome-wide methylome of human cells infected with a bacterial pathogen. For our study we have used human macrophages activated with interferon gamma, because we believe that this more accurately reflects the state of macrophages during sepsis caused by *B. pseudomallei*. We found that a range of genes associated with the immune response is differentially methylated in the host after infection with *B. pseudomallei* and these changes were replicated in a second experiment. These methylation events were associated with genes encoding cytokines, chemokines and their receptors (*CCR4*, IL1R2, IL18RAP, *TNFSF15*, *TNFSF8*, *CCL28*) and signalling pathways (*CASP8AP2*, *TOLLIP*, *SYK*, *ZBP1*, *MAP4K4*, *MBIP*). Immune system processes were also enriched in genes near iDMPs identified in this study and genes differentially expressed in patients’ blood infected with *B. pseudomallei*. Increased expression of the *TOLLIP* gene has previously been reported in humans infected with *B. pseudomallei*^6^. The precise role of *TOLLIP* in protective immunity is still being clarified. One function of the protein is to interact with *IL1RI*, *TLR2* and *TLR4* after lipopolysaccharide (LPS) activation, thereby modulating the NF-κB and JNK signaling pathways and consequently inflammatory responses to infection^20^. The role of *B. pseudomallei* LPS in virulence^21^ and the activation of *TLR2* and *TLR4* signalling during *B. pseudomallei* infection is well documented^22^,^23^,^24^. It is possible that the differential methylation of *TOLLIP* we have seen modulates the early immune response to *B. pseudomallei* LPS.

Another important feature of disease caused by *B. pseudomallei* is the ability of the pathogen to establish chronic or persistent infections. *B. pseudomallei* is an intracellular pathogen, and phagocytes are believed to be an important niche for growth and survival in the host^25^,^26^. The differential methylation of genes involved in memory T-cell responses, such as *CD44*, *FOXO1* and *FOXC1*, might contribute to the inability of the host to mount responses capable of clearing infection. Other host defence systems involve interferon gamma and nitric oxide. Interferon gamma plays a key role in the control of *B. pseudomallei* infection^27^,^28^ and suppression of the host innate response to *B. pseudomallei* infection has previously been attributed to downregulation of the type I interferon gamma signalling pathway by the bacterial effector TssM^29^. Previously CD44^high^ CD8 T-cells have been shown to be an important source of interferon gamma^30^ and we found evidence of differential methylation of *CD44* in our study. We also found evidence of the differential methylation of a number of genes (*IRF-8*, *IFNE* and *ZBP1*), which are involved in the regulation of interferon gamma expression. Nitric oxide has potent antibacterial activity towards *B. pseudomallei*^31^,^32^. A DMP located upstream of the dimethylarginine dimethylaminohydrolase 2 (*DDAH2*) gene that encodes an enzyme in the nitric oxide generation pathway is hypo-methylated at all time points. The differential methylation of the gene encoding the *NOX4 NADPH* oxidase that we have seen, might explain the activation of this enzyme in phagocytes infected by the bacterium^33^. Such observations provide new insights in the possible molecular mechanisms, which underpin suppression of the host response.

We also found differential methylation of genes associated with ubiquitination of proteins targeted for degradation, including the *SPSB4* and *WSB1* gene products, which are associated with substrate recognition and the *NEDD4L* and *SIAH1* genes encoding ubiquitin ligases. The deamidation of the *NEDD8L* protein by a *B. pseudomallei* type III effector protein (*CHBP*) has previously been demonstrated, and this triggers apoptosis of host cells^34^. However, the modulation of host cell ubiquitination has also been associated with the suppression of host immunity^35^ and the downregulation of the NF-kappaB/type I interferon signalling pathways has been attributed to the ubiquitination of signalling molecules^29^. Our findings provide additional insight into the mechanisms that contribute to modulation of the ubiquitination pathway.

The most significantly enriched GO terms in the set of iDMPs were cell adhesion related (FDR corrected P < 0.05). *B. pseudomallei* is known to promote the polymerisation of host actin at one pole of the bacterium in an *ARP2/3*- dependent manner^36^. This enables motility of the bacteria and is essential for the uptake of bacteria into cells and the spread of bacteria within and between cells. Several iDMPs map to genes involved in actin binding or polymerisation. These include *CDC42BPA*, a component of the BLOC-1 complex, which can interact with *WASH* - a regulator of *ARP2/3*^37^ and *ACTR3*, a major component of the *ARP2/3* complex^38^. We also found differential methylation of one of the central regulators of actin polymerisation, the *PAK1* p21-activated kinase^39^.

Our findings also suggest that there are some common targets for methylation in cells infected with pathogens. Most of the methylation changes in cells infected with *M. tuberculosis* or *B. pseudomallei* are losses rather than gains in methylation. A broadly similar trend has also been reported by Marr *et al*.^15^ who reported a larger proportion of hypo-methylated CpG sites in *L. donovani* infected macrophages. Overall it seems that there is a general trend toward demethylation of host cell DNA during infection. Our comparison of *M. tuberculosis* or *B. pseudomallei* induced changes in methylation patterns revealed that although a number of common genes were differentially methylated, there were no conserved iDMPs. However, since these studies used different cell types we cannot discount the possibility that there would be conserved iDMPs if the experimental conditions were identical.

We also found that the conserved iDMPs were significantly enriched in the first exon of genes, DNA methylation in this genomic compartment has been associated with transcriptional suppression^40^,^41^, suggesting that a large proportion of the infection associated DNA methylation changes observed could be altering gene transcription.

In summary, our work provides new insights into the extent to which infection with a bacterial pathogen results in differential methylation of host cell DNA. Our findings indicate that methylation of host DNA occurs on a much greater scale than previously suggested. None of the effector *B. pseudomallei* molecules identified to date have been shown to methylate host DNA. Whether differential methylation is a consequence of the direct interaction of bacterial methyltransferases with host cell DNA, or the consequence of modification of host cell methyltransferase activity awaits investigation.

## Methods

### Cell line and infection model

The human leukemic monocyte lymphoma cell line (U937, ATCC CRL-1593.2) was maintained in RPMI 1640 supplemented with 10% foetal bovine serum (FBS) at 37 °C. U937 cells were differentiated to macrophage-like cells following exposure to 20 ng/ml (final concentration) of phorbol 12-myristate 13-acetate (PMA) for 48 hours at 37 °C and differentiation evidenced by increased adherence to tissue culture flasks. A total of eight flasks (four for infected cells and four for uninfected controls) were prepared.

Overnight cultures of *B. pseudomallei* K96243 were diluted in L-15 medium and added to differentiated U937 cells at a multiplicity of infection (MOI) of 10. Where indicated *B. pseudomallei* K96243 expressing red fluorescent protein (RFP) was used^42^. Uninfected controls in the remaining four flasks were overlaid with L15 medium only. The cells were then incubated at 37 °C for 2h to allow infection. The cells were washed 3 times with phosphate buffered saline (PBS) and incubated with fresh L15 medium containing 1 mg/ml kanamycin for 2 hr to kill extracellular bacteria. After 2 hrs the macrophage cells were held in fresh media containing 250 μg/ml kanamycin to supress the growth of extracellular bacteria. At appropriate time points the cells were washed 3 times in warm PBS and lysed with 0.1% (vol/vol) Triton X-100. DNA was isolated using an AllPrep kit (Qiagen) and stored at −80 °C until required. DNA yield was measured using a Nanodrop instrument with measurements between 22.8–50.6 ng/ul.

### Enumeration of adhesion and uptake of *B. pseudomallei* by U937 cells

At 1 hour (T1), 2 hours (T2), 4 hours (T4) and 24 hours (T24) post infection the cells were washed 3 times in warm PBS and lysed with 0.1% (vol/vol) triton X-100. Serial dilutions of the cell lysate were plated onto LB agar to determine the intracellular bacterial cell counts (Fig. 2).

Additionally at T2, cells were washed 3 times with PBS and overlaid with 200 μl paraformaldehyde 0.4%, ensuring any coverslips were fully immersed. Cells were than incubated at room temperature for 30 minutes. PFA was removed and coverslips were washed twice with PBS for 1 hour for each wash. Coverslips were removed and stained with 4′,6-diamidino-2-phenylindole (DAPI) and cells visualized with epifluorescence. Eight fields of view were visualised and the number of uninfected or infected U937 cells counted. The mean number of cells associated with bacteria at T2 was calculated (Fig. 1).

A second experiment was designed to enable verification of the results of the first experiment. Similar procedures were followed as described above and DNA collected from uninfected and infected cells at 1 hour (T1), 2 hours (T2), 3 hours (T3) and 4 hours (T4) post infection.

### DNA methylation analysis

The DNA methylation profile of the infected macrophage DNA was determined using the Infinium HumanMethylation450 BeadChip (450K) (Illumina Inc.) following the manufacturer’s instructions. The 450 k BeadChip interrogates DNA methylation at >480,000CpG sites across the genome. Briefly, 500 ng of genomic DNA was sodium bisulfite converted using the EZ-96 DNA Methylation kit (Zymo research, CA, USA) using the manufacturer’s instructions. Post-hybridisation allelic-specific single-base extension of the probes incorporates a fluorescent label enabling detection. Replicates were processed together on the same array to reduce batch effects. The data was extracted and the initial analysis was performed using GenomeStudio (2010.3) methylation module (1.8.5). For further details please see Pidsley *et al*.^43^.

### Data Analysis

For each CpG site a beta value was generated by the relative intensity of the green fluorescent signal (methylated (M)) to the red and green signal combined (M/M+U+100). Quality control checks and quantile normalisation were implemented using WateRmelon^43^. Samples with more than 1% of sites with a detection p-value greater than 0.05 were removed as were probes with 1% of samples with a detection p-value greater than 0.05. Probes were removed if they had a bead count less than 3 in 1% of samples. Cross-hybridizing probes were removed^44^, leaving 425496 probes for analysis. DMPs between infected and control cells were identified using Δβ cut-off of 0.1. iDMPs identified at T2 and T4 in the first experiment, were compared to the corresponding probes at the same time point in the second experiment. Probes exhibiting a Δβ change in the same direction of =>0.1 were taken for further analysis. Correlation of Δβ values between the two experiments was measured using Pearson Correlation Coefficient. This set of replicated iDMPs were tested for enrichment of genomic regions using a two-tailed Fisher’s exact test, compared to the frequency of all probes on the Illumina 450 K array. iDMPs with consistent up or down-methylation throughout T1, T2, T3 and T4 were determined. The temporal methylation patterns were assigned to categories based on the change in score at successive time points. As many of the differential methylation patterns found in disease or environmental factors are characterized by smaller changes, in the range of 5%^15^, this criterion was selected to identify temporal patterns of differential methylation. Signatures with more than 5% differential methylation at all time points were classified as, constant hyper- (+5%) or constant hypo-methylated (−5%) probes. Signatures with more than 5% differential methylation at early time points (T1, T2, T3) and no change at the T4 time point were classified as early response probes. Signatures with no response at the T1 time point and with more than 5% differential methylation at later time points were classified as late response probes. Signatures with no response at T1 and T4 and more than 5% differential methylation at T2 or T3 were classified as transient responses. The remainder of the patterns were classified as oscillatory probes.

Candidate genes were assigned to the probes using the GREAT software^45^, genes are allotted to genomic regions taking into account the functional significance of *cis*-regulatory regions. Gene ontology term enrichment analysis was performed using the Bioconductor package GOseq^46^. Enriched gene ontology terms are selected based on the criteria of having p-value < 0.05. Redundant gene ontology terms were removed and the non-redundant gene ontology terms were clustered using semantic similarity measures (the *simRel* score) using REVIGO^47^.

## Additional Information

**Accession codes**: All of the DNA methylation data are available at the GEO database (accession number GSE83379).

**How to cite this article**: Cizmeci, D. *et al*. Mapping epigenetic changes to the host cell genome induced by *Burkholderia pseudomallei* reveals pathogen-specific and pathogen-generic signatures of infection. *Sci. Rep*. **6**, 30861; doi: 10.1038/srep30861 (2016).

## Supplementary Material

Supplementary Information

## Figures and Tables

**Table 1:** Number of iDMPs assigned to categories based on differential methylation patterns.

Category	Number of probes	Hyper-methylated	Hypo-methylated
A: constant response	21	3	18
B: early response	23	10	13
C: late response	55	11	44
D: transient response	99	42	57
E: oscillatory response	190	NA	NA

Signatures with differential methylation at all time points (1 hour (T1), 2 hours (T2), 3 hours (T3) and 4 hours (T4) post infection) were classified as, constant hyper- or constant hypo-methylated probes. Signatures with differential methylation at early time points (T1, T2, T3) and no change at the T4 time point were classified as early response probes. Signatures with no response at the T1 time point and with differential methylation at later time points were classified as late response probes. Signatures with no response at T1 and T4 and differential methylation at T2 or T3 were classified as transient responses. The remainder of the patterns were classified as oscillatory probes.

NA = not applicable.
