# Supplementary material for: Mapping epigenetic changes to the host cell genome induced by Burkholderia pseudomallei reveals pathogen-specific and pathogen-generic signatures of infection
Source: Sci Rep. 2016 Aug 3;6:30861. doi: 10.1038/srep30861 (PMC4971488; doi:10.1038/srep30861)
Supplement: Supplementary Information [file srep30861-s1.pdf]

Mapping epigenetic changes to the host cell genome induced by *Burkholderia pseudomallei* reveals pathogen-specific and pathogen-generic signatures of infection.

Deniz Cizmeci[1,+], Emma L. Dempster[2,+], Olivia L. Champion[3], Sariqa Wagley[3], Ozgur E. Akman[1], Joann L. Prior[3], Orkun S. Soyer[4,*], Jonathan Mill[2,5,*], Richard W. Titball[3,*].

[1] College of Engineering, Mathematics and Physical Sciences, University of Exeter, Exeter, United Kingdom

[2] University of Exeter Medical School, Exeter University, Exeter, United Kingdom

[3] College of Life and Environmental Sciences, University of Exeter, Exeter, United Kingdom

[4] School of Life Sciences, University of Warwick, United Kingdom

[5] Institute of Psychiatry, Psychology & Neuroscience, King's College London, United Kingdom

[+]These authors contributed equally to this work

[*]Corresponding authors

Supporting Information

Supplementary Figure 1. The proportion (74%) of cells infected at T2 determined by microscopy.

Supplementary Figure 2. Experimental design.

Supplementary Table 1. Replicated infection induced differentially methylated probes (iDMPs). (A) iDMPs replicating at T2. (B) iDMPs replicating at T4.

Supplementary Figure 3. Methylation changes in experiments 1 and 2, at replicated iDMPs. (A) Changes at T2. (B) Changes at T4.

Supplementary Table 2. iDMPs by genomic feature

Supplementary Figure 4. Methylation levels in *M. tuberculosis* and *B. pseudomallei* infections at conserved iDMPs in *B. pseudomallei* infection. (A) *B. pseudomallei* infection at T2. (B) *B. pseudomallei* infection at T4.

Supplementary Table 3. Comparison with publically available DNA methylation changes during infection with M. tuberculosis. Genes annotated to the iDMPs in *B. pseudomallei* and in *M. tuberculosis* infections.

Supplementary Table 4. Comparison with publically available transciption data. Genes annotated to conserved iDMPs in our study and differentially expressed in patients with septisemic melioidosis.

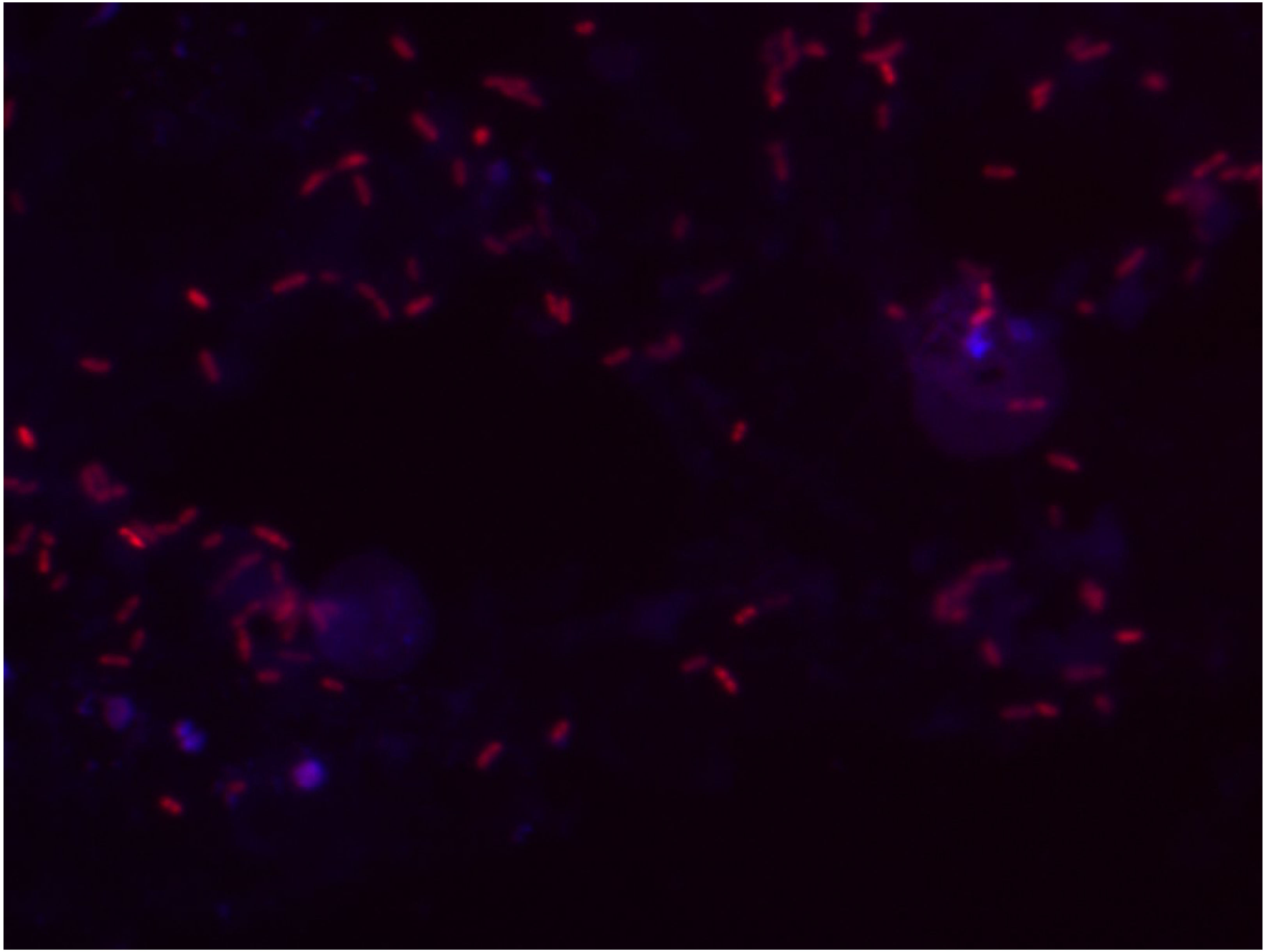

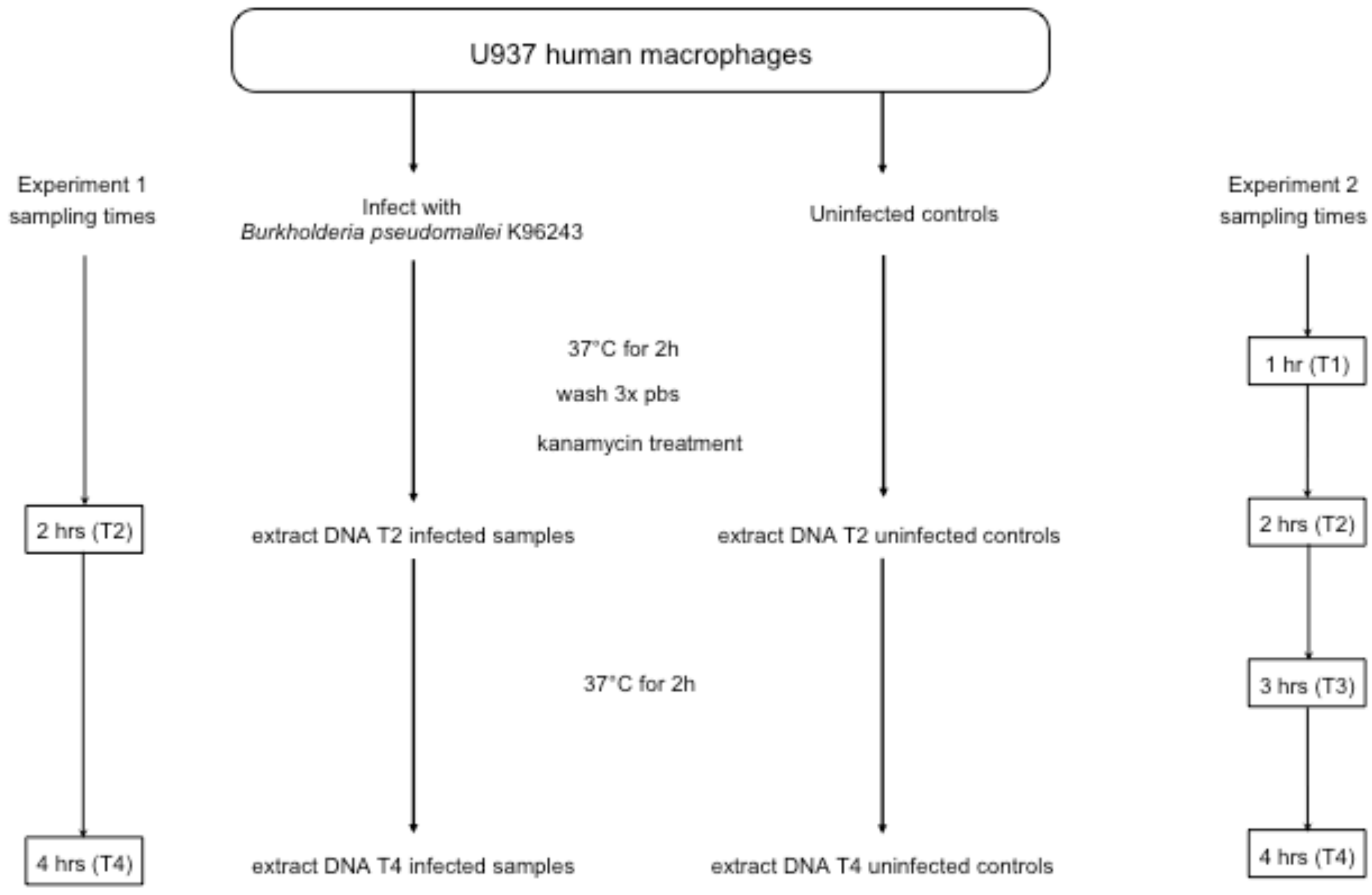
U937 human macrophages
Experiment 1
sampling times
Infect with
Burkholderia pseudomallei K96243
Uninfected controls
Experiment 2
sampling times
37°C for 2h
wash 3x pbs
kanamycin treatment
1 hr (T1)
2 hrs (T2)
extract DNA T2 infected samples
extract DNA T2 uninfected controls
2 hrs (T2)
37°C for 2h
3 hrs (T3)
4 hrs (T4)
extract DNA T4 infected samples
extract DNA T4 uninfected controls
4 hrs (T4)

a

b

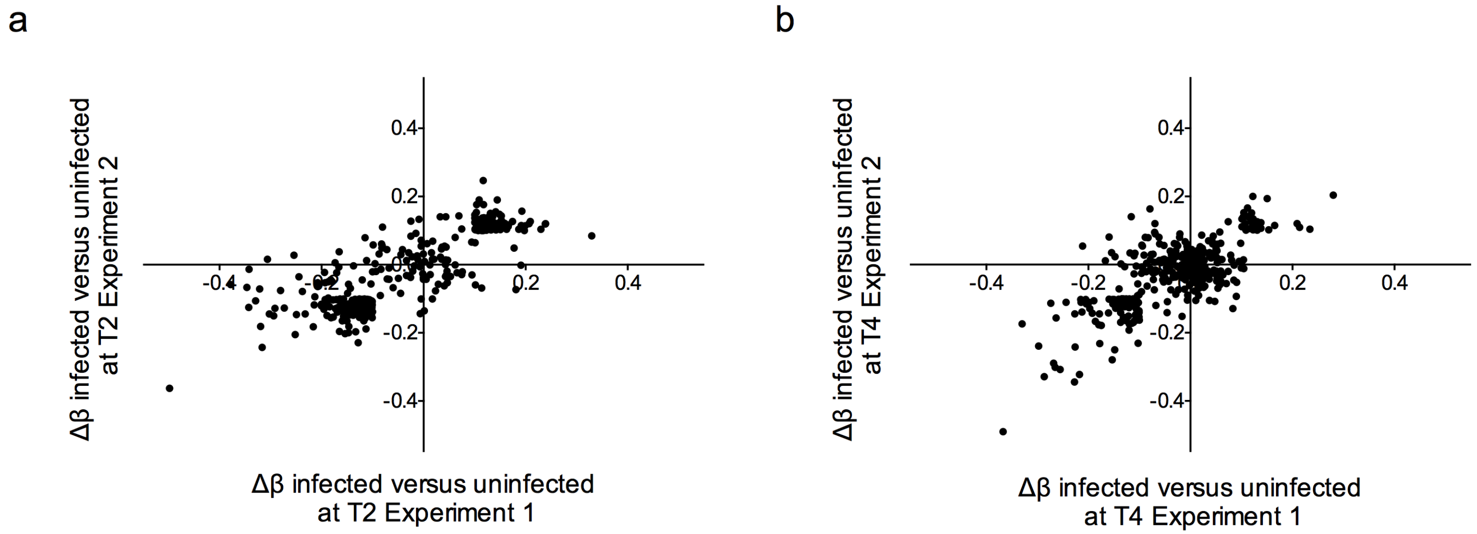
Δβ infected versus uninfected
at T2 Experiment 2
Δβ infected versus uninfected
at T2 Experiment 1
0.4
0.2
-0.2
-0.4
-0.4
-0.2
0.2
0.4
Δβ infected versus uninfected
at T4 Experiment 2
Δβ infected versus uninfected
at T4 Experiment 1

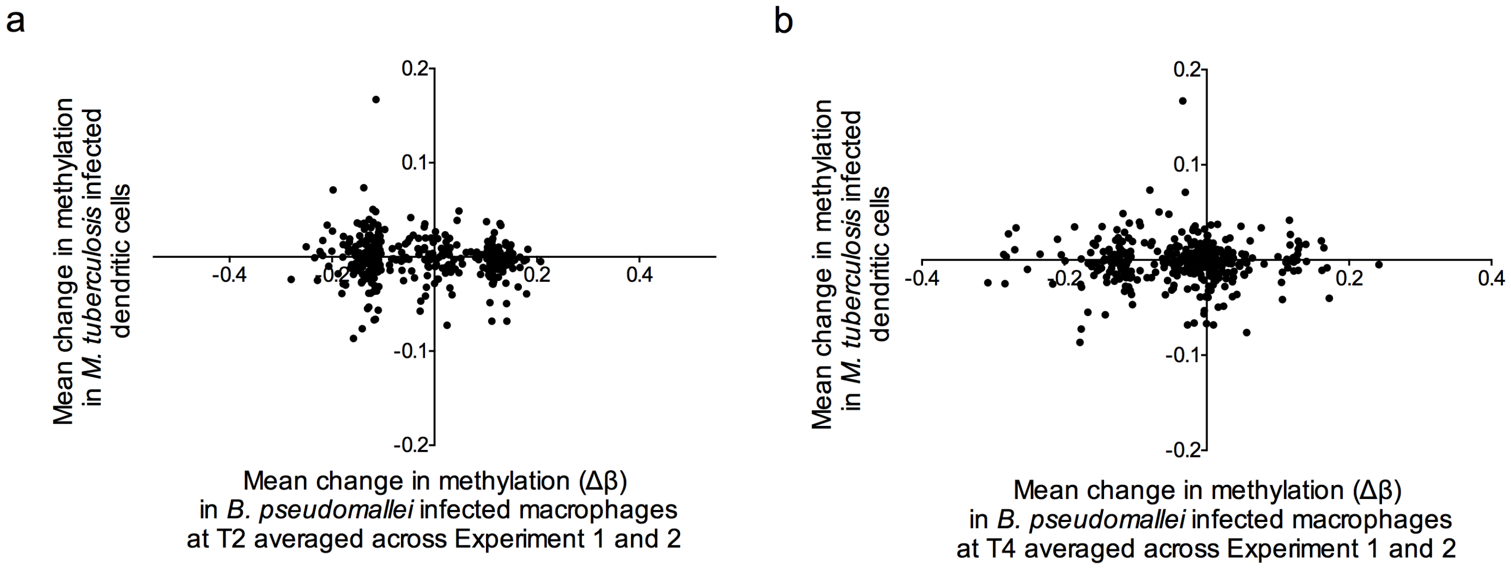
a
0.2
0.1
-0.1
-0.2
-0.4
-0.2
0.2
0.4
Mean change in methylation
in *M. tuberculosis* infected
dendritic cells
Mean change in methylation (Δβ)
in *B. pseudomallei* infected macrophages
at T2 averaged across Experiment 1 and 2
b
0.2
0.1
-0.1
-0.2
-0.4
-0.2
0.2
0.4
Mean change in methylation
in *M. tuberculosis* infected
dendritic cells
Mean change in methylation (Δβ)
in *B. pseudomallei* infected macrophages
at T4 averaged across Experiment 1 and 2

Supplementary Table 1. (A) Replicated infection induced differentially methylated probes (iDMPs) at T2 in infected versus uninfected human macrophages.

Methylation sites and annotation										Methylation level differences (Δβ)						
Probe	Gene annotation using GREAT	Chromosome (chr)	Position	UCSC REFGENE NAME	UCSC REFGENE GROUP	UCSC CPG ISLANDS NAME	RELATION TO UCSC CPG ISLAND	Transcription Factor Binding Site Annotation	DHS Annotation	Δβ T2 infected versus uninfected Experiment 1	Δβ T4 infected versus uninfected Experiment 1	Δβ T1 infected versus uninfected Experiment 2	Δβ T2 infected versus uninfected Experiment 2	Δβ T3 infected versus uninfected Experiment 2	Δβ T4 infected versus uninfected Experiment 2	**Differential methylation pattern**
cg00048832	GABRA1 (-2760)	5	161274631	GABRA1	TSS1500			NRSF	1	0.11717813	0.03438222	-0.004404	0.24730848	-0.0448397	-0.0187379	transient response hypermethylation
cg07938480	OR1F2P (-333)	16	3265228	OR1F2P	TSS1500			NA	0	0.14438473	0.00016213	-0.053704	0.19016031	0.02443694	-0.0330175	oscillatory response
cg18831045	CDC25C (-474)	5	137667989	CDC25C	TSS1500;TSS1500	chr5:137667502-137668037	Island	Pol2|HEY1|HDAC2_	0	0.10938888	-0.0951329	0.03132455	0.18977628	-0.0129569	-0.0069658	transient response hypermethylation
cg00758915	PCDHGC5 (-95268), TAF7 (-73189)	5	140773539	PCDHGA4	Body	chr5:140773855-140774441	N_Shore	NA	0	0.10448559	0.0511167	0.10033766	0.17685855	0.02970327	-0.019112	early response hypermethylation
cg27621528	GPC1 (+17503), ANKMY1 (+104787)	2	241392617	PP14571;GP(	Body;Body	chr2:241391876-241393598	Island	Pol2	0	0.11781393	-0.1058362	0.19486726	0.17622012	-0.1305379	-0.1199211	oscillatory response
cg04133410	PTCH1 (-1127)	9	98271957	PTCH1;PTCH	TSS1500;Body;Body	chr9:98272643-98273383	N_Shore	HA-E2F1|Ini1	1	0.19250076	0.0707561	0.02616392	0.15754276	0.03533834	-0.0551208	oscillatory response
cg21860629	LOR (+2090), PGLYRP3 (+48925)	1	153234268	LOR	Body			NA	0	0.14119877	-0.0103378	-0.0797033	0.1557244	-0.0537203	-0.0436497	oscillatory response
cg10648547	PASK (+37110), SNED1 (+113554)	2	242051808	PASK	Body			NA	0	0.10361568	-0.0466902	-0.0842519	0.15372391	0.06048216	-0.0091283	oscillatory response
cg27359566	ZFHX3 (-15901)	16	73098174			chr16:73099812-73100791	N_Shore	BAF155|Ini1|Pol2|S	1	0.13136858	0.02813195	0.0827783	0.15060233	0.05084654	-0.0055484	early response hypermethylation
cg12450347	TRIM67 (-122695), ARV1 (+61156)	1	231175978	FAM89A;FAN	5'UTR;1stExon	chr1:231175062-231176317	Island	HMGN3|CCNT2|Pol	1	0.13260386	-0.1544563	0.03544483	0.14944668	-0.0782559	0.03533962	oscillatory response
cg22829908	MLLT10 (-9107), C10orf140 (-7716)	10	21814466	C10orf140	5'UTR	chr10:21814786-21815716	N_Shore	Pol2|HDAC2_(SC-62	1	0.14890323	0.01721991	-0.0210929	0.14808498	-0.0019222	0.02236006	transient response hypermethylation
cg20429250	SLFN12L (+398)	17	33814359	SLFN12L	Body	chr17:33814234-33814947	Island	Pol2|SUZ12|Egr-1|Y	1	0.10048839	-0.0687821	-0.0058796	0.14666333	-0.1130169	0.09002075	oscillatory response
cg13499210	DMRT2 (-55040), DMRT3 (+18616)	9	995579			chr9:998463-999034	N_Shelf	NA	0	0.15241536	-0.0210323	0.2256893	0.14317592	0.03266068	0.08060678	oscillatory response
cg11359849	NAV3 (-506491), E2F7 (-259218)	12	77718577			chr12:77718453-77719440	Island	CTCF|ZNF263|Rad2	1	0.1352172	0.01389413	0.00346657	0.14050885	-0.0208686	0.00759996	transient response hypermethylation
cg14097425	MTAP (-243083), IFNE (-77240)	9	21559551	LOC554202	Body	chr9:21559133-21559816	Island	Pol2-4H8|Pol2	0	0.12670867	-0.0881664	-0.0279221	0.13853684	0.06558034	0.08028452	late response hypermethylation
cg10445453	ADAMTS14 (-18)	10	72432540	ADAMTS14;A	TSS200;TSS200	chr10:72432242-72432738	Island	TBP|E2F6|CCNT2|H	0	0.12978727	-0.073589	-0.0281042	0.13851049	0.00926703	-0.0233771	transient response hypermethylation
cg05599550	ABCC3 (-315)	17	48711902	ABCC3;ABCC	TSS1500;TSS1500	chr17:48712050-48712705	N_Shore	USF-1|JunD|TBP	1	0.10195109	0.05960762	-0.0046199	0.13766617	0.00134074	-0.0477932	transient response hypermethylation
cg16919456	ARHGAP28 (+95359), LAMA1 (+188022)	18	6929790			chr18:6929450-6930088	Island	E2F6|Pol2|E2F6_(H-	0	0.11850093	-0.0485952	-0.0317444	0.13759857	0.01898528	-0.04157	transient response hypermethylation
cg25759517	PYCR1 (-39)	17	79895006	PYCR1;PYCR1	TSS200;TSS200	chr17:79894668-79895533	Island	Pol2|Pol2-4H8	0	0.11531041	-0.0208348	-0.0584653	0.13739596	-0.0011366	-0.0317144	oscillatory response
cg00886554	NMU (-134)	4	56502598	NMU	TSS200	chr4:56501912-56502523	S_Shore	ZNF263|HMGN3|HA	0	0.10044123	-0.0769912	0.05489736	0.13738244	0.02651348	0.02976454	early response hypermethylation
cg17351085	RAB3IL1 (-261)	11	61685257	RAB3IL1	TSS1500	chr11:61684591-61685216	S_Shore	Pol2|CCNT2|HA-E2F	1	0.11593012	0.01717758	-0.0216726	0.13434846	-0.0561155	-0.036464	oscillatory response
cg11249313	DOCK10 (-277)	2	225907606	DOCK10	TSS1500	chr2:225906653-225907464	S_Shore	NA	1	0.10755443	-0.0051723	-0.0378797	0.13281484	-0.0567207	0.01905603	oscillatory response
cg24479328	SYT1 (+277250), PAWR (+368107)	12	79716682	SYT1;SYT1;SY	Body;Body;Body			NA	0	0.11859595	0.01993023	-0.027586	0.13271221	-0.0559396	-0.0087045	oscillatory response
cg08780020	KIAA1826 (+73)	11	105892880	KIAA1826;KI	1stExon;5'UTR	chr11:105892725-105892952	Island	Pol2|Pol2-4H8|TAF1	1	0.11653229	-0.0028006	-0.0803286	0.13166866	-0.055295	0.02041831	oscillatory response
cg11422541	ADCY6 (-4710)	12	49182586	ADCY6	5'UTR	chr12:49182421-49182658	Island	CtBP2	1	0.1025179	0.05299174	-0.0316565	0.131506	0.00882527	0.05969353	oscillatory response
cg17629148	B4GALNT2 (-96732), IGF2BP1 (+38823)	17	47113596	IGF2BP1;IGF	Body;Body	chr17:47113256-47113490	S_Shore	CTCF|YY1_(C-20)|Ra	1	0.15273946	-0.0241888	-0.0169474	0.13031161	-0.0103382	-0.0294323	transient response hypermethylation
cg14701410	SLC4A8 (+88)	12	51818681	SLC4A8;SLC4	5'UTR;1stExon;5'UTR	chr12:51818460-51819166	Island	HA-E2F1|SP1|Nrf1|	1	0.12747347	-0.0706298	-0.055611	0.12857304	-0.1357131	-0.0733548	oscillatory response
cg18453320	FOXC1 (+5429), GMDS (+629758)	6	1616109			chr6:1604606-1615866	S_Shore	NA	1	0.12124919	-0.0720421	0.08740539	0.12846703	0.02386151	-0.024473	early response hypermethylation
cg11630696	ROBO1 (-275)	3	79817333	ROBO1	TSS1500	chr3:79815638-79815900	S_Shore	USF-1	0	0.1041885	-0.0073323	0.07984858	0.12756229	0.0908929	0.02593736	early response hypermethylation
cg13959031	GOLM1 (-181831), ISCA1 (+1149)	9	88896340	ISCA1	Body	chr9:88896741-88897721	N_Shore	NA	1	0.17437589	-0.0143422	-0.0118611	0.12715938	0.06327891	0.02995745	transient response hypermethylation
cg12077581	PANX2 (-14)	22	50609145	PANX2	TSS200	chr22:50609181-50609627	N_Shore	Pol2|YY1_(C-20)|E2	1	0.20949518	0.08578358	0.00858517	0.12707931	0.0205424	-0.0009785	transient response hypermethylation
cg26127652	ZNF311 (-392)	6	28973426	ZNF311	TSS1500			E2F6|HA-E2F1|CCN	1	0.1047759	0.00999849	-0.0012336	0.12508459	0.02251254	0.03874274	transient response hypermethylation
cg10982775	FKBP14 (-1525), PLEKHA8 (-184)	7	30067792	PLEKHA8	TSS1500	chr7:30067762-30068600	Island	Pol2|Pol2-4H8|TBP|	1	0.10584576	-0.0429999	0.00913856	0.12471956	-0.0199121	0.00260347	transient response hypermethylation
cg06123396	ADAM23 (-745)	2	207307622	ADAM23	TSS1500	chr2:207308266-207308907	N_Shore	CTCF|Rad21|CTCF_(	1	0.13736652	-0.1100788	0.03384137	0.12459717	0.03256633	0.02645456	transient response hypermethylation
cg20783424	OR6B3 (-90794), OTOS (+3790)	2	241076282	MYEOV2	TSS1500	chr2:241075403-241075916	S_Shore	USF-1	0	0.13143364	0.05016444	-0.1086484	0.1245402	0.06621623	0.04656665	oscillatory response
cg05917480	SIAH1 (+49308), LONP2 (+91710)	16	48369920	LONP2	Body			NA	1	0.1005494	0.04056847	0.06610939	0.12414076	-0.0054632	0.01943434	early response hypermethylation
cg21241989	DMRT1 (-2563)	9	839126			chr9:841374-843364	N_Shelf	NA	0	0.15490349	0.03694635	-0.0760828	0.12385576	0.09321498	-0.0297705	oscillatory response
cg21084674	IGFL2 (+54)	19	46651553	IGFL2;IGFL2	5'UTR;1stExon			NA	0	0.13711127	-0.0403403	0.03225464	0.12321111	-0.0139034	0.00378373	transient response hypermethylation
cg01493198	SF3B14 (-247)	2	24299560	SF3B14	TSS1500	chr2:24300060-24300294	N_Shore	Pol2|ZEB1_(SC-2538	1	0.15622208	-0.0692783	-0.0149065	0.1227938	0.03188912	0.12026438	oscillatory response
cg08341316	DKK2 (+497)	4	107956955	DKK2;DKK2	1stExon;5'UTR	chr4:107956555-107957453	Island	NA	1	0.14555732	0.09590551	-0.0173348	0.12155373	-0.072205	0.01008008	oscillatory response
cg19187155	NMUR1 (-88)	2	232395269	NMUR1	TSS200	chr2:232394763-232395337	Island	CTCF|ELF1_(SC-631)	1	0.15892428	-0.1477815	0.00935544	0.12146488	-0.1348922	0.02335672	oscillatory response
cg01458686	NAV3 (-506232), E2F7 (-259477)	12	77718836			chr12:77718453-77719440	Island	ZNF263|HA-E2F1|T	0	0.10649671	0.06403109	0.00390909	0.12130652	0.04765915	-0.030135	transient response hypermethylation
cg18429742	ZDHHC11 (-1)	5	851101	ZDHHC11	TSS200	chr5:848389-848770	S_Shelf	NA	0	0.23930446	0.05147398	0.05675269	0.12029914	0.08628339	-0.0478795	early response hypermethylation
cg17400701	GBX2 (+237384), AGAP1 (+436535)	2	236839267	AGAP1;AGAF	Body;Body			NA	0	0.20673595	0.02194219	-0.0506624	0.12013028	-0.0587245	-0.0097848	oscillatory response
cg04134305	SHH (-828554), RNF32 (-2150), C7orf13 (-	7	156433520	C7orf13;RNF	TSS200;5'UTR	chr7:156432433-156433670	Island	Pol2|AP-2gamma|E	0	0.23877902	0.0211124	-0.0508012	0.11940984	-0.000888	0.03273807	oscillatory response
cg17288747	IYD (-142073), PPP1R14C (+83767)	6	150547954	PPP1R14C	Body			NA	0	0.11020717	-0.0353755	0.05489467	0.1193003	0.03472265	-0.0226266	early response hypermethylation
cg01451328	SIX1 (+1101), SIX6 (+139116)	14	61115053	SIX1	Body	chr14:61114102-61116552	Island	ZNF263|CTCF	1	0.1662911	0.01576835	0.01962551	0.11672381	-0.0457923	-0.033998	transient response hypermethylation
cg12280664	USP6NL (+256486), CELF2 (+257895)	10	11317787	CUGBP2	Body			NA	0	0.13458108	-0.1061998	0.03037292	0.11509041	0.08853634	-0.0039188	transient response hypermethylation
cg22496973	GCFC2 (+575)	2	75937535	C2orf3	Body	chr2:75937657-75938139	N_Shore	NA	1	0.11029574	-0.0683844	-0.0549359	0.11493467	-0.0406286	-0.0156716	oscillatory response
cg09755105	GBX1 (-121)	7	150864987	GBX1	TSS200	chr7:150863966-150865511	Island	NA	0	0.19145795	-0.0534381	-0.0806392	0.11491735	0.10754752	0.0109314	oscillatory response
cg16048803	KRT28 (-627)	17	38956837	KRT28	TSS1500	chr17:38953213-38953513	S_Shelf	PU.1	0	0.1135446	0.05218424	0.00162398	0.11469089	0.00524277	0.0405527	transient response hypermethylation
cg01641792	PDE4A (+41030), KEAP1 (+41691)	19	10572362	PDE4A	Body			Pol2|TAF1|E2F6_(H-	1	0.19918438	0.08106196	-0.034215	0.1145581	0.11806525	0.01322841	transient response hypermethylation

cg21966319	PALLD (+124910), CBR4 (+388341)	4	169543126	PALLD	Body			p300|FOXA1_(C-20)	1	0.11508487	-0.0699105	0.10473865	0.11453772	0.09493343	0.09499057	constant hypermethylation
cg09247297	LPHN2 (-322434)	1	81943647					NA	1	0.11982751	-0.0088022	0.03147702	0.11407225	-0.0133453	0.01208146	transient response hypermethylation
cg18030105	MRGPRE (-142622), ZNF195 (+4214)	11	3396237	ZNF195	Body	chr11:3400155-3400419	N_Shelf	NA	0	0.11763961	-0.0063021	0.05274889	0.11294097	-0.0428181	0.05411252	oscillatory response
cg00782124	KIF13B (-85908), DUSP4 (+1749)	8	29206517	DUSP4;DUSP	TSS200;Body	chr8:29205664-29209847	Island	AP-2alpha|AP-2gam	1	0.11514697	0.00784729	0.01836722	0.11276324	-0.0063993	0.00122052	transient response hypermethylation
cg18799217	TMEM161B (-420925), MEF2C (+214332)	5	87985589			chr5:87985470-87985810	Island	NANOG_(SC-33759)	1	0.11705066	0.01305151	-0.0202127	0.11261346	0.01129624	-0.0431408	transient response hypermethylation
cg11280288	PIK3R4 (+3560), COL6A6 (+182958)	3	130462135	PIK3R4	Body	chr3:130465478-130465700	N_Shelf	NA	0	0.11436854	-0.1033761	-0.0019844	0.11260043	0.01290173	-0.0912962	oscillatory response
cg06482188	RBFOX3 (-165866), ENPP7 (-60336)	17	77644545					NA	1	0.14215359	0.04245372	-0.0001969	0.1121966	-0.0453547	0.0323716	transient response hypermethylation
cg11147094	NTRK3 (+379523)	15	88420438	NTRK3	Body			NA	0	0.10449757	0.01955806	-0.0474369	0.11212299	-0.0081489	0.02300104	transient response hypermethylation
cg02130133	ANO5 (-918)	11	22213803	ANO5	TSS1500	chr11:22214172-22215320	N_Shore	CTCF	0	0.15897302	-0.0102156	-0.0047968	0.11210595	-0.0225546	0.06773421	oscillatory response
cg10639888	RPRM (-47)	2	154335368	RPRM	TSS200	chr2:154334449-154335458	Island	HA-E2F1|ZNF263|T	1	0.10392143	-0.0060367	-0.0042461	0.11201817	0.03395487	0.03180807	transient response hypermethylation
cg27326226	GJB2 (-665)	13	20767778	GJB2	TSS1500	chr13:20766208-20767779	Island	NA	0	0.13825075	-0.0446411	-0.0566395	0.1119594	-0.0407747	0.01259072	oscillatory response
cg14403184	VENTX (-2199)	10	135049208			chr10:135048797-135052077	Island	NA	0	0.16582941	-0.1184315	0.01727683	0.11187574	-0.0587289	0.03411174	oscillatory response
cg25767906	SCP2 (-119)	1	53392781	SCP2	TSS200	chr1:53392880-53393645	N_Shore	NA	1	0.10956586	-0.1590412	-0.0144609	0.11119118	-0.0654989	0.08082446	oscillatory response
cg25234159	EHHADH (+100992), VPS8 (+340963)	3	184870893	C3orf70	TSS200	chr3:184870185-184871525	Island	E2F6_(H-50)	1	0.19531481	0.01569547	-0.0205583	0.11117176	-0.0952454	0.04271342	oscillatory response
cg23902471	FAM19A5 (+153714)	22	49039001	FAM19A5	Body	chr22:49042371-49042632	N_Shelf	NA	0	0.10720375	-0.1158517	-0.0914825	0.11069543	-0.0263197	0.14079928	oscillatory response
cg01793593	ROBO1 (+749621)	3	79067437	ROBO1	Body	chr3:79067882-79068566	N_Shore	NA	0	0.1255185	-0.0158687	-0.0367076	0.10982667	0.02886514	-0.0222637	transient response hypermethylation
cg11011938	SEMA5A (+256)	5	9545976	SEMA5A;SEM	5'UTR;1stExon	chr5:9544692-9546715	Island	HA-E2F1	0	0.11074852	0.00266551	0.02715105	0.10978637	-0.0467778	-0.0141865	transient response hypermethylation
cg05143078	EDC3 (+329)	15	74988056	EDC3	5'UTR	chr15:74988166-74988669	N_Shore	NA	0	0.12681228	-0.0843618	-0.0061417	0.10911236	-0.0101186	-0.0627996	oscillatory response
cg13688098	AASDH (+71782), CEP135 (+366819)	4	57181855	KIAA1211	Body	chr4:57180957-57182147	Island	NA	0	0.14737407	0.08535652	0.00172098	0.10877485	0.02021454	-0.0452023	transient response hypermethylation
cg24716125	NBEAL2 (-87364), PTH1R (+10070)	3	46933808	PTH1R	Body	chr3:46933746-46934293	Island	Max|c-Myc	1	0.11944255	-0.0856585	0.12193495	0.10876139	-0.0164962	0.00367744	early response hypermethylation
cg01525017	DNAJC19 (-474649), SOX2 (-247501)	3	181182210					NA	0	0.10701948	-0.0549836	0.00874737	0.10828913	-0.0024598	-0.0282988	transient response hypermethylation
cg21806985	MCC (-120)	5	112824646	MCC	TSS200	chr5:112823256-112824304	S_Shore	NA	1	0.15074165	-0.0055048	-0.040137	0.10815469	-0.0299739	0.04394213	transient response hypermethylation
cg23415761	GNPDA1 (-176)	5	141392795	GNPDA1	TSS200	chr5:141392360-141392587	S_Shore	HA-E2F1|Pol2|ZNF2	1	0.11796612	-0.0519216	0.0003326	0.10782949	-0.0025122	0.0566334	oscillatory response
cg15526246	MSRA (-151402), TNKS (+346983)	8	9760427	LOC157627	Body	chr8:9760750-9761643	N_Shore	NA	0	0.10969093	-0.0367815	0.04701419	0.10756229	0.05265922	0.02933215	transient response hypermethylation
cg14406859	TRPV2 (-4673)	17	16314182			chr17:16310306-16310914	S_Shelf	NA	0	0.11923228	0.02863457	-0.194545	0.10711642	-0.08993	-0.0628707	oscillatory response
cg02666008	RBM15 (-1578)	1	110880366			chr1:110880394-110880624	N_Shore	CEBPB|CCNT2|BAF1	1	0.13431501	-0.0285335	-0.0515531	0.10627811	-0.0341944	-0.0864078	oscillatory response
cg02016838	ZC3H12C (-555)	11	109963531	ZC3H12C	TSS1500	chr11:109963240-109964677	Island	HA-E2F1|Pol2-4H8|	1	0.14950264	-0.0702292	0.0473032	0.10598467	-0.0164502	0.0068095	transient response hypermethylation
cg01276381	LATS1 (-376)	6	150039767	LATS1	TSS1500	chr6:150038664-150039440	S_Shore	eGFP-GATA2|GATA-	1	0.12140868	-0.0005005	-0.0198535	0.10543173	-0.0725089	-0.0302889	oscillatory response
cg05559643	NDNF (-144024), QRFPR (+164484)	4	122137696	TNIP3	TSS200			NA	1	0.11316021	0.02837039	-0.0235968	0.1052502	0.10526024	-0.0192178	transient response hypermethylation
cg06570432	SORCS2 (+106803), PSAPL1 (+135523)	4	7301176	SORCS2	Body			NA	1	0.11197437	-0.0062105	0.00499488	0.10486896	0.01614314	0.05336272	oscillatory response
cg16792014	XPOT (-514364), SRGAP1 (+45248)	12	64283788	SRGAP1	Body			GR	1	0.10115394	0.02248623	-0.005828	0.10452102	-0.0851907	0.0863394	oscillatory response
cg20740029	SLC5A8 (+180)	12	101603835	SLC5A8;SLC5	1stExon;5'UTR	chr12:101603387-101603933	Island	HA-E2F1|SUZ12|USI	0	0.16303252	-0.0113407	0.00578367	0.10444761	-0.0304109	-0.0717856	oscillatory response
cg06918804	PRIMA1 (-51)	14	94254816	PRIMA1	TSS200	chr14:94253952-94255733	Island	TBP|HA-E2F1	1	0.22991434	0.02732713	-0.0026148	0.10441179	0.00479842	-0.0241899	transient response hypermethylation
cg26215849	MTRF1 (+49)	13	41837663	MTRF1;MTRI	5'UTR;1stExon			HA-E2F1|Pol2|NFKB	1	0.18649884	0.01647275	-0.0246899	0.10431898	-0.2337346	-0.090723	oscillatory response
cg18947032	KIAA1279 (-202)	10	70748274	KIAA1279	TSS1500	chr10:70748435-70749334	N_Shore	Mxi1_(bHLH)|IRF3|	0	0.12575394	0.01867021	-0.1636914	0.10390616	-0.0719273	0.03160601	oscillatory response
cg10195962	AGAP9 (-442744), ANXA8L2 (-90550)	10	47656369			chr10:47655677-47656379	Island	ZNF263|CHD2_(N-1	1	0.12893798	0.05254257	-0.0264808	0.10388994	0.02663847	-0.0421191	transient response hypermethylation
cg14194478	FCN2 (-148564), COL5A1 (+90442)	9	137624093	COL5A1	Body			NA	0	0.12398908	-0.0040457	0.02370415	0.10382819	0.04887971	0.00377586	transient response hypermethylation
cg06824157	TRIO (+313673), ANKH (+414385)	5	14457501	TRIO	Body	chr5:14461130-14461463	N_Shelf	NA	0	0.1241481	0.03579466	0.06326067	0.10308786	-0.038654	0.06968213	oscillatory response
cg00868860	DCD (+200)	12	55041948	DCD	Body			NA	0	0.10088246	0.0462961	-0.0701135	0.1029584	-0.0094968	-0.0160133	oscillatory response
cg23346462	PEX5L (-17)	3	179754533	PEX5L	TSS200	chr3:179754520-179755245	Island	GATA3_(SC-268)|NR	1	0.15066387	0.0423836	0.0041519	0.10282447	-0.0245305	0.07007786	oscillatory response
cg00985958	B3GAT2 (+60791), SMAP1 (+228518)	6	71605996	B3GAT2	Body			NA	0	0.101464	-0.001559	-0.0131252	0.10272479	0.07507657	-0.0611707	oscillatory response
cg16059311	INSIG1 (-106943), HTR5A (+119997)	7	154982542					CTCF|HA-E2F1|CCN	1	0.102524	0.01367106	0.010984	0.10257361	-0.0165656	-0.0093312	transient response hypermethylation
cg12816057	ADRB2 (-171949), HTR4 (-17583)	5	148034206	HTR4	TSS1500	chr5:148033472-148034080	S_Shore	PU.1	1	0.10604515	0.11194423	-0.1518773	0.10233968	-0.0232675	0.1668183	oscillatory response
cg19797516	PDSS1 (-130140), APBB1IP (+129189)	10	26856454	APBB1IP	3'UTR	chr10:26855906-26856198	S_Shore	NA	0	0.1103772	-0.0101005	0.01127834	0.10220574	0.03915784	-0.0033016	transient response hypermethylation
cg11125883	MDM1 (-2885)	12	68729045			chr12:68725847-68726475	S_Shelf	NA	0	0.11808638	0.00998759	0.01690148	0.10193381	0.01133192	0.01399226	transient response hypermethylation
cg23926793	KHDRBS2 (-565)	6	62996664	KHDRBS2	TSS1500	chr6:62995855-62996228	S_Shore	NA	0	0.14232482	-0.0204341	0.03329289	0.10189555	-0.0258383	0.09040897	oscillatory response
cg14051353	SPATA19 (-84850), IGSF9B (+26638)	11	133800241	IGSF9B	Body	chr11:133800684-133800931	N_Shore	NA	0	0.1323263	-0.0598925	-0.0145042	0.10184422	-0.0046336	0.06114465	oscillatory response
cg04752565	SMAGP (-454)	12	51664655	SMAGP;SMA	TSS1500;TSS1500	chr12:51663404-51664411	S_Shore	BAF170|Ini1|Pol2|H	1	0.10719587	-0.0267167	0.06518821	0.10174991	0.03492138	-0.00379	early response hypermethylation
cg04112471	ACPL2 (-167251), SPSB4 (+12688)	3	140783430	SPSB4	5'UTR	chr3:140784809-140785481	N_Shore	NA	0	0.11073176	0.02597585	-0.0453173	0.10088703	-0.0615812	-0.0666514	oscillatory response
cg27125304	PRKG1 (+212478), CSTF2T (+495966)	10	52963388	PRKG1;PRKG	Body;Body			NANOG_(SC-33759)	1	0.1158633	-0.0500059	-0.0085128	0.10085946	-0.0210171	-0.0381306	transient response hypermethylation
cg17966435	MYL5 (-4255), ATP5I (+671)	4	667455	ATP5I	Body	chr4:667273-667601	Island	Pol2|Pol2-4H8|HEY	1	0.12316134	-0.0615464	-0.1076778	0.10083742	-0.0633556	0.08039274	oscillatory response
cg18316974	GLMN (-182470), GFI1 (+4592)	1	92947035	GFI1;GFI1;GF	Body;Body;Body	chr1:92945907-92952609	Island	NA	1	0.10702721	-0.0166454	-0.0234539	0.10064875	0.02135241	-0.0007817	transient response hypermethylation
cg15371617	HAVCR2 (+110)	5	156536137	HAVCR2;HAV	5'UTR;1stExon			Pol2|TAF1|TBP|EBF	1	0.11386369	0.00791653	0.03874405	0.10037843	-0.0695179	-0.032499	oscillatory response
cg00032805	MARCH1 (-67)	4	165304473	MARCH1	TSS200	chr4:165304328-165305177	Island	Rad21|SUZ12	1	0.1169911	-0.2110892	-0.0835517	0.1001369	-0.0398041	0.05453259	oscillatory response
cg26947543	NONE	13	69459742					NA	0	0.1976014	-0.0059024	0.01093246	0.10007656	0.04046681	-0.011614	transient response hypermethylation
cg20984663	DDX39A (-13689), PKN1 (-7202)	19	14543883	PKN1	TSS1500	chr19:14543873-14544704	Island	E2F6|TAF1|ELF1_(S	1	-0.1253055	0.07922716	-0.0094579	-0.1000156	0.00549619	0.02757179	transient response hypomethylation
cg03821311	HIST1H1B (-660)	6	27836018	HIST1H1B	TSS1500	chr6:27835190-27835461	S_Shore	NA	1	-0.163567	0.03732775	0.05976245	-0.1000505	0.03665162	0.0353896	oscillatory response
cg19786805	LRRK1 (+133882), CHSY1 (+198795)	15	101593341	LRRK1	Body	chr15:101593447-101593882	N_Shore	NA	0	-0.156553	-0.0147107	0.05246169	-0.1001947	-0.0006784	-0.0385792	oscillatory response
cg25551699	FOXO1 (+169833), COG6 (+841137)	13	41070900					NA	0	-0.1159596	0.02801394	0.03050803	-0.1005856	0.05404218	0.03609146	oscillatory response
cg13702235	FNIP2 (-992)	4	159689189	FNIP2	TSS1500	chr4:159689700-159690823	N_Shore	NA	0	-0.119635	0.05804469	-0.038242	-0.1008728	0.00745948	0.09554956	oscillatory response
cg13810101	FAT1 (-782980), ZFP42 (-488958)	4	188427966			chr4:188426310-188426593	S_Shore	NA	0	-0.1108866	-0.068088	0.05968446	-0.1009221	0.04300387	-0.0217875	oscillatory response
cg14652646	LDB2 (+52579), BC010030 (+619559)	4	16847844	LDB2	Body			NA	1	-0.1341124	0.04527951	-0.0201184	-0.1009269	0.0123592	-0.0247837	transient response hypomethylation
cg03067367	ALG1 (+171730)	16	5293539			chr16:5289582-5290386	S_Shelf	NA	0	-0.1272453	0.05269976	-0.0433807	-0.1019919	-0.0095492	0.00606445	transient response hypomethylation

cg13476313	NR5A1 (+24934), GPR144 (+31342)	9	127244764	NR5A1	3'UTR			IRF1	1	-0.1710666	0.05516015	-0.0083572	-0.1020914	0.02887434	-0.0284706	transient response hypomethylation
cg25711289	CRIPAK (-48932), MAEA (+52736)	4	1336407			chr4:1340107-1341471	N_Shelf	NA	0	-0.1075282	-0.040113	0.03654529	-0.1021332	-0.0673356	-0.0076708	transient response hypomethylation
cg05798436	DAPP1 (-164830), MTTP (+87911)	4	100573150			chr4:100573059-100573578	Island	NA	0	-0.1166537	-0.1104475	0.04465304	-0.1021976	0.12257471	-0.0274308	oscillatory response
cg16013919	DNAJB6 (+529163), PTPRN2 (+721609)	7	157658872	PTPRN2	Body	chr7:157659433-157660322	N_Shore	NA	0	-0.1894816	0.03673274	-0.0224007	-0.1023748	0.07014649	-0.0423302	oscillatory response
cg10958087	HINT1 (+190072)	5	130310961					NA	0	-0.1930255	0.01249791	0.0316184	-0.1024771	0.06968627	0.02447629	oscillatory response
cg19373290	NKX6-2 (-138762), TTC40 (+17790)	10	134738298			chr10:134738218-134738704	Island	NA	0	-0.1055146	-0.0394131	0.00655938	-0.1028567	0.02275378	0.00957523	transient response hypomethylation
cg03018678	IRX5 (-612769), IRX3 (-31964)	16	54352341					NA	0	-0.1773215	-0.0291179	0.02984008	-0.103027	-0.0543989	-0.0365671	transient response hypomethylation
cg09795569	DYRK3 (+720)	1	206809600	DYRK3;DYRK	5'UTR;Body	chr1:206808523-206809187	S_Shore	Pol2|POU2F2|Oct-2	1	-0.1902969	-0.0126362	0.10060173	-0.1041695	0.11281928	-0.022789	oscillatory response
cg10599998	STARD4 (-208764), NREP (+255707)	5	111056920					NA	0	-0.1122786	-0.0541279	0.02297788	-0.1041927	0.04126604	-0.044418	transient response hypomethylation
cg14045860	CD44 (-2133)	11	35158283			chr11:35160375-35161000	N_Shelf	CEBPB	0	-0.1216759	-0.1395371	0.09196881	-0.1043405	0.13781118	-0.071275	oscillatory response
cg17374364	OTP (+5457), PDE8B (+422359)	5	76929064	OTP	Body	chr5:76932317-76933523	N_Shelf	NA	0	-0.1394528	0.05749992	-0.01177	-0.104453	0.00334046	-0.0590038	oscillatory response
cg11579999	ZNF295 (-1034)	21	43431529	ZNF295	TSS1500	chr21:43429279-43431489	S_Shore	Rad21|CTCF|CTCF_(	1	-0.1192012	0.01794743	-0.0169923	-0.1045898	0.03998002	-0.0413715	transient response hypomethylation
cg08822075	NFE2L3 (+1761), HNRNPA2B1 (+46805)	7	26193607	NFE2L3	Body	chr7:26191794-26192757	S_Shore	Pol2|TAF1|POU5F1_	0	-0.123512	-0.0167127	-0.0079141	-0.1047667	0.13731887	0.02167449	oscillatory response
cg25828427	E2F7 (-412583), NAV3 (-353126)	12	77871942					NA	0	-0.1292445	-0.0446526	-0.0033378	-0.1048479	0.08573781	-0.1089076	oscillatory response
cg13430807	SF3B4 (-3172)	1	149903315	MTMR11	Body	chr1:149899625-149899893	S_Shelf	NA	0	-0.1127565	0.00357478	0.04539782	-0.105317	-0.0332489	0.03149004	transient response hypomethylation
cg23079522	PPM1L (+95633), B3GALNT1 (+253054)	3	160569628	PPM1L	Body			NA	1	-0.1152649	0.03163082	0.00297724	-0.1059424	-0.0485587	-0.0200865	transient response hypomethylation
cg03972398	HOXC13 (+604)	12	54333179	HOXC13	1stExon	chr12:54332805-54333731	Island	NA	1	-0.1255517	0.11864824	-0.0589809	-0.1060001	0.04847834	0.12587368	oscillatory response
cg18610738	METTL7B (-240)	12	56075089	METTL7B	TSS1500			CTCF|Rad21|SMC3_	1	-0.1071326	0.01268175	-0.0846663	-0.106171	-0.0394062	0.00572923	early response hypomethylation
cg22937556	TUBA3C (+63656)	13	19692335	DKFZp686A1	Body			NA	0	-0.1020467	0.07423393	0.12005529	-0.1061957	-0.0308433	0.12604221	oscillatory response
cg14159894	NONE	20	11872261	BTBD3	5'UTR	chr20:11871374-11872207	S_Shore	HA-E2F1|Pol2|Egr-1	1	-0.3291896	-0.0010868	0.02539884	-0.1062475	-0.0376078	-0.0225841	transient response hypomethylation
cg23452498	PRELID2 (+325291)	5	144889607					NA	0	-0.1184982	0.05090374	0.04521313	-0.1066622	0.01192907	0.02248068	transient response hypomethylation
cg18114671	NOL4 (-322)	18	31803836	NOL4	TSS1500	chr18:31804480-31804875	N_Shore	NA	0	-0.1250888	-0.0453046	-0.0313733	-0.1073588	-0.0861059	-0.0185581	transient response hypomethylation
cg00994876	D2HGDH (-10786), ING5 (+21788)	2	242663243	ING5	Body	chr2:242663547-242663872	N_Shore	NA	0	-0.1003092	-0.0272545	0.04698225	-0.1078962	-0.1096416	0.06042687	oscillatory response
cg05110803	PGPEP1L (+163561), IGF1R (+192563)	15	99385323	IGF1R	Body			NA	0	-0.1219528	-0.0508452	0.05878879	-0.1080538	0.04833663	0.01573245	oscillatory response
cg11741189	PAX6 (-4378)	11	31843886			chr11:31841315-31842003	S_Shore	NA	0	-0.1084196	0.00968486	-0.0098192	-0.1092474	-0.1029489	-0.008542	transient response hypomethylation
cg11670211	NOX4 (+364)	11	89224288	NOX4;NOX4	5'UTR;Body	chr11:89224416-89224718	N_Shore	NA	1	-0.1582207	0.09843061	0.03846049	-0.1092509	0.07242737	0.06266525	oscillatory response
cg13210581	KCNA4 (+393761)	11	29644815					Pol2|TBP|SP1|TAF1	1	-0.1250909	0.03079794	0.03290753	-0.1095618	-0.0241474	-0.0257262	transient response hypomethylation
cg23644960	TRPC6 (-165)	11	101454823	TRPC6	TSS200	chr11:101453357-101454684	S_Shore	NA	1	-0.1193003	-0.0789419	0.04764188	-0.1107341	0.00922545	0.16363132	oscillatory response
cg06596075	CAPS2 (-4917), GLIPR1L1 (+290)	12	75728752	GLIPR1L1	Body			NA	1	-0.1560511	0.0250721	-0.0440867	-0.1109223	-0.0855369	-0.0423432	transient response hypomethylation
cg16454107	TMEM100 (+772)	17	53799452	TMEM100	5'UTR			NA	0	-0.1083643	0.00588544	0.0305869	-0.1110197	-0.0257634	-0.0135591	transient response hypomethylation
cg00400832	DLX5 (+3819), DLX6 (+15034)	7	96650323	DLX5	Body	chr7:96650221-96651551	Island	NA	0	-0.1009333	0.01548216	0.04205927	-0.1112511	0.06596688	-0.0585979	oscillatory response
cg01343624	COBL (-457606)	7	51842120					USF-1|CTCF|Rad21	1	-0.1248942	0.09098739	0.01909155	-0.1113721	-0.0464002	-0.0516207	oscillatory response
cg10102131	MLLT4 (+1036), HGC6.3 (+148912)	6	168228706	C6orf124;ML	TSS1500;Body	chr6:168226063-168228559	S_Shore	Pol2	1	-0.1913237	-0.1083968	0.01977064	-0.1116416	-0.0053975	0.03303157	transient response hypomethylation
cg25667495	NR1I2 (-76380), C3orf15 (+3308)	3	119425176	C3orf15	Body	chr3:119421855-119422334	S_Shelf	NA	0	-0.1398935	0.00295177	-0.0036579	-0.1118229	-0.0596785	0.03606098	transient response hypomethylation
cg02235871	C3orf23 (-317937), ABHD5 (+329632)	3	44062006			chr3:44063314-44063837	N_Shore	NA	0	-0.1018694	-0.0570376	0.05352547	-0.1120131	0.06748186	0.00606904	oscillatory response
cg00899976	ZBP1 (-31909), PMEPA1 (+57490)	20	56227540	PMEPA1	Body	chr20:56227252-56227687	Island	NA	0	-0.1366346	0.01583857	-0.0682228	-0.1122131	-0.0561895	-0.0213323	early response hypomethylation
cg00309339	TOMM7 (-31233), FAM126A (+160116)	7	22893653			chr7:22893794-22894577	N_Shore	Pbx3|CTCF|AP-2gan	0	-0.1192003	-0.0433078	-0.006468	-0.1122492	0.04124836	0.00763709	transient response hypomethylation
cg24674640	MLL5 (-25886), LHFPL3 (+659647)	7	104628750			chr7:104624337-104624848	S_Shelf	NA	1	-0.1240198	-0.0050673	-0.0423293	-0.1122624	0.00510236	-0.0896944	oscillatory response
cg16857428	ZNF559 (+759)	19	9435206	ZNF559;ZNF5	1stExon;5'UTR	chr19:9434784-9435144	S_Shore	TFIIIC-110	0	-0.1137592	0.00668074	-0.0205497	-0.1123011	0.05521317	0.01682529	oscillatory response
cg27112993	LHX1 (-51966), MRM1 (+284781)	17	35242805					KAP1	1	-0.1066903	0.02296979	-0.0167114	-0.1123511	-0.0259051	-0.0312706	transient response hypomethylation
cg15392054	RER1 (-51548), SKI (+111532)	1	2271665	MORN1	Body			ZNF263	0	-0.131629	-0.0673479	0.01638509	-0.1131828	0.0030162	-0.1196669	oscillatory response
cg00705142	PVRL3 (-817)	3	110789788	PVRL3	TSS1500	chr3:110790149-110791401	N_Shore	CTCF	1	-0.1212591	0.02229784	-0.0039231	-0.1132423	-0.0031518	-0.012172	transient response hypomethylation
cg13782919	SYK (+24831), AUH (+535363)	9	93588842	SYK;SYK;SYK	Body;5'UTR;TSS1500			NA	1	-0.1024156	0.00816401	0.00379497	-0.1134949	-0.0219484	-0.0776868	oscillatory response
cg12934804	ARG2 (-29)	14	68086549	ARG2	TSS200	chr14:68086277-68086957	Island	CtBP2|E2F6|JunD|c	1	-0.1184776	0.04485049	-0.0508911	-0.1135438	0.07784009	0.0839542	oscillatory response
cg07309764	GNAI1 (-225)	7	79763914	GNAI1	TSS1500	chr7:79763793-79764889	Island	CtBP2|Ini1|GR|CTCI	1	-0.1890427	-0.0907129	0.03433562	-0.1142243	-0.0443624	0.02810088	transient response hypomethylation
cg18934293	MEST (-332)	7	130131566	MEST;MEST;	TSS1500;5'UTR;TSS15	chr7:130130739-130133111	Island	NA	0	-0.1133929	0.03735805	-0.0889657	-0.114537	-0.1025383	-0.1047447	constant hypomethylation
cg13180315	HOXD3 (-4510)	2	177024294			chr2:177024501-177025692	N_Shore	SUZ12	0	-0.1472264	0.09961299	-0.0205668	-0.1146531	0.00190856	-0.0095693	transient response hypomethylation
cg04598517	SLC6A3 (0)	5	1445542	SLC6A3;SLC6	5'UTR;1stExon	chr5:1444678-1446648	Island	Pol2	1	-0.1175989	0.02719035	0.03330908	-0.1148982	-0.0238902	0.01264521	transient response hypomethylation
cg15586341	ANAPC16 (-75959), SPOCK2 (-51268)	10	73899798	ASCC1	Body			NA	0	-0.1052366	0.0022459	-0.0615627	-0.115026	-0.0764757	0.01691061	early response hypomethylation
cg15886307	CIT (-575)	12	120315669	CIT	TSS1500	chr12:120314847-120315169	S_Shore	NA	0	-0.1220969	0.05974475	-0.0294468	-0.1151903	0.05332925	0.06258451	oscillatory response
cg27543214	DDR1 (-94636), IER3 (-49502)	6	30761828					p300|TBP|FOXA1_(	1	-0.1250874	-0.1359157	-0.0003136	-0.1152452	0.06123619	0.00206338	oscillatory response
cg13706365	USP6NL (+70054), CELF2 (+444327)	10	11504219	USP6NL;USP	3'UTR;3'UTR	chr10:11505222-11505766	N_Shore	CEBPB	0	-0.1424812	-0.0084425	0.1092866	-0.1152484	0.01080463	0.01251892	oscillatory response
cg09256131	BCL11B (-672)	14	99738493	BCL11B;BCL1	TSS1500;TSS1500	chr14:99739801-99740920	N_Shore	Pol2|CTCF|Pol2-4H8	0	-0.1243757	-0.0117715	-0.0218644	-0.1153548	-0.0181553	-0.0028174	transient response hypomethylation
cg05848045	SOX4 (+176762), PRL (+532348)	6	21770733	FLJ22536	Body			NA	0	-0.1211589	-0.0726906	0.06395654	-0.1158776	-0.0297167	-0.0433587	oscillatory response
cg13333304	CLDN11 (-452)	3	170136200	CLDN11	TSS1500	chr3:170136242-170137886	N_Shore	NA	1	-0.2000531	-0.1666905	-0.0616191	-0.1161261	0.01223893	0.01179875	early response hypomethylation
cg21617762	UBE3C (-120137), MNX1 (-8171)	7	156811517			chr7:156810572-156814593	Island	NA	0	-0.1622636	0.0598507	-0.0069056	-0.1162208	0.01029419	0.01492085	transient response hypomethylation
cg02426376	ALG6 (-39273), FOXD3 (+5258)	1	63793987			chr1:63795363-63796140	N_Shore	NA	1	-0.1285353	0.01423357	0.06175506	-0.116388	-0.0325055	-0.0582488	oscillatory response
cg19512521	SLC22A17 (-243)	14	23821902	SLC22A17;SL	5'UTR;1stExon;TSS15	chr14:23821087-23822106	Island	CTCF	0	-0.1003361	-0.0957706	0.02756562	-0.1175567	-0.0353068	-0.0255813	transient response hypomethylation
cg00135497	LRAT (-3313)	4	155661849			chr4:155661678-155661984	Island	NA	1	-0.2142357	-0.0730995	0.03957393	-0.118125	0.03930401	0.03110602	transient response hypomethylation
cg06340987	DBX1 (-826)	11	20182695	DBX1	TSS1500	chr11:20184570-20185497	N_Shore	SUZ12	0	-0.1560561	0.0598027	0.00212493	-0.119252	-0.0188953	-0.0189528	transient response hypomethylation
cg13861948	DUSP10 (+405752), HLX (+457021)	1	221509763	LOC400804	TSS200			PRDM1_(Val90)	1	-0.1103591	0.00132379	-0.02041	-0.1194131	0.02092904	0.00991769	transient response hypomethylation
cg13897122	SLC9A4 (-50219), IL18RAP (+4289)	2	103039542	IL18RAP	5'UTR			NA	1	-0.1193771	0.03714251	0.0877889	-0.1199142	-0.0028821	-0.0254156	oscillatory response
cg11814312	DNAH17 (-98681), CYTH1 (+106219)	17	76672156	CYTH1;CYTH	3'UTR;3'UTR			NA	0	-0.1085978	-0.0957571	0.04615675	-0.1199185	-0.029414	-0.0792774	oscillatory response

cg20411555	MKLN1 (-412519), KLF14 (-181188)	7	130600075					NA	0	-0.1999601	-0.0236835	-0.0227137	-0.1203684	0.02960376	-0.073699 oscillatory response
cg04606367	FEZF1 (-1649)	7	121946213			chr7:121945345-121946235	Island	SUZ12	0	-0.1171646	0.04066557	-0.0064107	-0.1208545	0.02379042	0.02720827 transient response hypomethylation
cg24258592	RAB11FIP2 (-770)	10	119806883	CASC2;RAB1:	Body;TSS1500;Body	chr10:119805825-119806544	S_Shore	HA-E2F1	0	-0.1277204	-0.0349975	0.0800081	-0.1209218	-0.0029915	0.02789413 oscillatory response
cg03706720	EPHB3 (-48648), CHRD (+133078)	3	184230938			chr3:184231301-184231924	N_Shore	NA	0	-0.1155099	0.02205356	-0.0243225	-0.1212447	-0.0963804	0.02302159 transient response hypomethylation
cg05059922	PLXNC1 (-298002), CRADD (+173346)	12	94244496	CRADD	3'UTR			NA	0	-0.1033248	-0.0057833	0.04420872	-0.1216673	0.04695587	-0.0227967 transient response hypomethylation
cg05984044	GABRB1 (+887)	4	47034181	GABRB1	Body	chr4:47034427-47034940	N_Shore	NA	0	-0.1211603	0.01675783	-0.0235694	-0.121752	-0.0380331	-0.0693351 oscillatory response
cg25922624	TRIM2 (-50126), FHDC1 (+211337)	4	154075471	TRIM2	5'UTR	chr4:154074383-154075045	S_Shore	p300|TCF4|ERalpha	1	-0.1073702	-0.0863025	-0.0359934	-0.122164	-0.0175981	-0.0202592 transient response hypomethylation
cg22511684	ABCE1 (-450849), HHIP (+1159)	4	145568306	HHIP	Body	chr4:145566242-145567413	S_Shore	TAL1_(SC-12984)|Pc	1	-0.1021047	-0.0605028	0.01692559	-0.1224574	0.02165363	0.01238452 transient response hypomethylation
cg14321777	BNIP3 (-79375), JAKMIP3 (-43503)	10	133874809					NA	0	-0.1360941	-0.0778552	-0.0730218	-0.1225532	0.03673837	-0.0127099 early response hypomethylation
cg18115064	MDGA2 (+538)	14	48143618	MDGA2	Body	chr14:48143433-48145589	Island	NA	1	-0.1081727	-0.02655	-0.0560513	-0.1225841	0.06252307	0.02404231 oscillatory response
cg23806621	BHLHE23 (-115)	20	61638501	BHLHE23	TSS200	chr20:61636171-61639055	Island	NA	0	-0.1840408	-0.1859071	-0.177053	-0.1232239	-0.2505889	-0.1667438 constant hypomethylation
cg18061847	HAS2 (-699)	8	122654328	HAS2;HAS2A	TSS1500;Body	chr8:122651665-122652389	S_Shore	HA-E2F1	1	-0.1602161	-0.042394	0.06329251	-0.1239281	0.03971027	-0.0492945 oscillatory response
cg17164345	PAK1 (-86207), AQP11 (-29365)	11	77271314					BAF155|STAT3|GTF:	1	-0.3426322	-0.1482034	-0.0575908	-0.1252396	-0.1919325	-0.2498506 constant hypomethylation
cg23749448	RAB11FIP4 (-42284), EVI2A (-27591)	17	29676357	NF1;NF1	Body;Body	chr17:29672335-29672612	S_Shelf	NA	0	-0.1348511	-0.0024129	0.04931475	-0.1259077	0.02410539	0.02068137 transient response hypomethylation
cg05101231	TEX264 (-111830), RAD54L2 (+17864)	3	51593459	RAD54L2	5'UTR			NA	0	-0.1014348	-0.02777	0.09134515	-0.1259472	-0.0812769	0.03040528 oscillatory response
cg10820158	PCTP (-17526), TMEM100 (-10589)	17	53810813	TMEM100	TSS1500			NA	0	-0.1576482	0.03761439	-0.0728956	-0.1259577	-0.0052653	0.02490936 early response hypomethylation
cg03044684	HUNK (+2405), MIS18A (+403343)	21	33248032	HUNK	Body	chr21:33244910-33246609	S_Shore	NA	1	-0.1682911	-0.0338676	0.04285502	-0.1262562	0.09403194	0.01213326 oscillatory response
cg15965301	SEC14L1 (-31172), MGAT5B (+241035)	17	75105832					CTCF	1	-0.1374939	0.00405159	0.12457599	-0.1270084	0.05417472	-0.0527019 oscillatory response
cg06597095	CCL28 (-67874), C5orf28 (+3630)	5	43480361	C5orf28	5'UTR	chr5:43483519-43484555	N_Shelf	NA	0	-0.2725851	-0.2259461	-0.0815063	-0.1270944	-0.2314245	-0.2418151 constant hypomethylation
cg23699324	CTNNA2 (+249)	2	79740308	CTNNA2;CTN	1stExon;5'UTR;5'UTR	chr2:79739696-79740243	S_Shore	Pol2	0	-0.1684762	0.08365542	-0.0340705	-0.1271249	0.04197392	-0.1292121 oscillatory response
cg15287806	ZMYND11 (+11861), DIP2C (+497813)	10	237794	ZMYND11	Body			KAP1|CEBPB	1	-0.1153414	0.0026626	0.00394767	-0.1272944	0.12347388	0.04425434 oscillatory response
cg16554447	TINAG (-114750), MLIP (+82208)	6	54058452	C6orf142	Body			c-Jun	1	-0.1087946	0.05013927	-0.0516031	-0.1274299	0.02257792	0.04925962 early response hypomethylation
cg19192256	PTGDR2 (+1729), CCDC86 (+12286)	11	60621714	GPR44	5'UTR	chr11:60619924-60621111	S_Shore	eGFP-GATA2|GATA-	0	-0.2911395	-0.2971841	-0.1309176	-0.1277124	-0.2426533	-0.2388153 constant hypomethylation
cg06938699	MUC6 (-1070)	11	1037775	MUC6	TSS1500	chr11:1036420-1036827	S_Shore	NA	0	-0.106701	-0.0317059	0.06969744	-0.1280397	-0.0096521	0.06190836 oscillatory response
cg10582827	SCAND3 (-29353), TRIM27 (+307303)	6	28584464			chr6:28583934-28584289	S_Shore	E2F6|E2F6_(H-50)|N	1	-0.1475107	-0.1032603	-0.0273317	-0.1283284	-0.0217115	-0.0560673 oscillatory response
cg10900526	DDHD1 (-348988), BMP4 (+452236)	14	53969033					NA	0	-0.1726106	-0.0380946	-0.0537929	-0.1285766	0.03509897	-0.0741273 oscillatory response
cg23051046	SGPP2 (-111747), PAX3 (-13860)	2	223177574			chr2:223176493-223177515	S_Shore	NFKB	1	-0.149717	0.01442884	-0.074091	-0.1288999	0.02230195	0.01439114 early response hypomethylation
cg15470658	LRP1 (-4140)	12	57518141			chr12:57521524-57522159	N_Shelf	CTCF|ELF1_(SC-631)	1	-0.1307489	-0.0657944	-0.1401773	-0.1294209	-0.1299817	-0.0188096 early response hypomethylation
cg00865927	FERMT2 (-3230)	14	53421044			chr14:53417108-53418339	S_Shelf	Pol2-4H8|TBP	0	-0.1414382	0.00514972	-0.0132768	-0.1302405	-0.0177914	-0.0399398 transient response hypomethylation
cg02913918	ZNF648 (+263237), CACNA1E (+314924)	1	181767609	CACNA1E	Body	chr1:181767493-181767893	Island	NA	0	-0.1152893	-0.0080931	-0.0949334	-0.1302535	0.01559454	0.01393946 early response hypomethylation
cg07230440	TMEM132D (+30999)	12	130357212	TMEM132D	Body			NA	1	-0.1953944	-0.0938457	-0.0138673	-0.1305956	0.00535157	-0.0067017 transient response hypomethylation
cg26872045	SLC27A6 (-391620), FBN2 (-35855)	5	127909589					NA	0	-0.1015047	-0.0092195	0.03766178	-0.1314783	-0.0099895	-0.0442652 transient response hypomethylation
cg09241022	FOXG1 (+18244)	14	29254530	C14orf23	Body	chr14:29254365-29255069	Island	NA	0	-0.1397673	-0.0830304	0.019269	-0.131572	-0.0823192	0.07892424 oscillatory response
cg14888916	STK3 (-113512), OSR2 (-5210)	8	99951420			chr8:99952020-99954686	N_Shore	NA	1	-0.1154455	-0.0953138	0.06076627	-0.1322516	0.00550212	0.05699317 oscillatory response
cg04023150	RNF220 (+2105), TMEM53 (+267034)	1	44873064	RNF220	5'UTR	chr1:44871109-44874047	Island	NA	1	-0.1016955	-0.0784494	-0.0776878	-0.1336879	-0.0173163	0.01395422 early response hypomethylation
cg08050748	TRIM9 (-469)	14	51562890	TRIM9;TRIM!	TSS1500;TSS1500	chr14:51560116-51562487	S_Shore	Max	1	-0.1018679	-0.0396462	0.00538707	-0.133974	0.00037802	0.03946331 transient response hypomethylation
cg26238975	DIO3 (-507025), RTL1 (-169479)	14	101520662	MIR134;MIR(	TSS1500;TSS1500;Body;TSS1500			NA	0	-0.1473181	-0.0213408	0.04733458	-0.1351321	0.05727727	-0.0078162 oscillatory response
cg10370599	HS3ST2 (-701)	16	22825158	HS3ST2	TSS1500	chr16:22824616-22826459	Island	NA	1	-0.1578716	0.02247318	-0.0077913	-0.1360105	-0.0368069	-0.0605593 oscillatory response
cg11930390	IFT81 (-330)	12	110561809	IFT81;IFT81;I	TSS1500;TSS1500;TSS	chr12:110562095-110562700	N_Shore	Max|E2F6|USF2|HE	1	-0.1001224	-0.0522505	-0.0622886	-0.1375773	-0.006767	-0.0506009 oscillatory response
cg11856334	LRRTM1 (-890877)	2	81422363			chr2:81421204-81422532	Island	NA	0	-0.168951	0.04346548	0.03178274	-0.1384249	0.00329921	0.0606608 oscillatory response
cg11044716	RFXAP (-124209), SERTM1 (+21081)	13	37269129	C13orf36	5'UTR			NA	0	-0.1285464	-0.0936524	0.00392748	-0.1386136	-0.0843271	0.05414343 oscillatory response
cg11626175	JARID2 (+118548), DTNBP1 (+298214)	6	15365074	JARID2	Body			Pol2	1	-0.1020581	-0.0244616	0.0359051	-0.1386856	-0.0031332	0.05560137 oscillatory response
cg04578894	ADAMTS12 (+49826), TARS (+401400)	5	33842297	ADAMTS12	Body			NA	1	-0.1260244	0.03669262	0.00457387	-0.1388436	0.07150658	-0.0262864 oscillatory response
cg14870242	SAMD4A (+317)	14	55034646	SAMD4A;SAI	1stExon;1stExon	chr14:55031826-55035030	Island	Pol2|Rad21|CTCF|C	1	-0.1214234	0.00952052	0.01546915	-0.139628	-0.0102211	0.01649242 transient response hypomethylation
cg04476125	BMP3 (-643715), FGF5 (+120662)	4	81308403	C4orf22	Body	chr4:81307471-81307952	S_Shore	NA	0	-0.1324204	0.01441541	0.01769489	-0.1405719	-0.1442293	-0.0385764 transient response hypomethylation
cg03258749	GABPB2 (-2674)	1	151040405	MLLT11	3'UTR	chr1:151042870-151043537	N_Shelf	NA	0	-0.111065	0.04340734	0.00100853	-0.1406474	0.10404805	0.02151623 oscillatory response
cg15734230	PEG3 (-258878), USP29 (-20537)	19	57610971			chr19:57610688-57610937	S_Shore	NA	0	-0.1151409	0.01904984	-0.0315959	-0.1409181	0.04340172	0.05301505 oscillatory response
cg19197744	NOV (+967)	8	120429518	NOV	Body	chr8:120428398-120429178	S_Shore	AP-2gamma|AP-2alp	1	-0.1329073	-0.005771	-0.0480376	-0.1411109	0.02805997	-0.0270509 transient response hypomethylation
cg24200107	TRIML1 (+143699)	4	189204296			chr4:189203714-189204474	Island	NA	1	-0.1143206	0.00484425	0.00658046	-0.1422126	-0.0028945	-0.0117483 transient response hypomethylation
cg17676428	TOLLIP (+16849), MUC5B (+69748)	11	1314042	TOLLIP	Body	chr11:1315467-1317115	N_Shore	NA	0	-0.2317528	-0.1114635	-0.083926	-0.1440593	-0.1765289	-0.169519 constant hypomethylation
cg04138591	FOXK1 (-373947)	7	4347982			chr7:4347707-4348017	Island	NA	0	-0.3024692	-0.2268826	-0.1835983	-0.1444321	-0.2971245	-0.3437743 constant hypomethylation
cg10636760	TTC34 (-198366), ACTRT2 (-33450)	1	2904595					NA	1	-0.1245733	0.01128504	0.02114244	-0.1447593	-0.0365465	-0.0345729 transient response hypomethylation
cg14476364	EDEM3 (+274462), TSEN15 (+428768)	1	184449578	C1orf21	Body			NA	1	-0.1116101	-0.0353035	-0.014847	-0.1447747	0.01834257	0.01853397 transient response hypomethylation
cg17099445	ENGASE (+7073), RBFOX3 (+400588)	17	77078091	ENGASE	Body			Egr-1	0	-0.2493199	0.0480405	0.0679971	-0.146228	-0.1432553	-0.1077634 oscillatory response
cg15491540	ISM1 (-56868), SPTLC3 (+155923)	20	13145549	SPTLC3	3'UTR			NA	0	-0.102199	-0.0063442	-0.0180945	-0.1465578	-0.024919	0.0599062 oscillatory response
cg26232417	HNF4A (-1422)	20	43028501	HNF4A;HNF4	TSS1500;Body;TSS1500;Body;TSS1500			NA	0	-0.1025577	0.09007677	0.06066088	-0.1467064	-0.0740333	-0.092943 oscillatory response
cg08194009	KSR1 (-91607), WSB1 (+86323)	17	25707428					ELF1_(SC-631)|CCNT	1	-0.1446632	-0.1364068	-0.0394462	-0.1471775	-0.1123327	-0.1691295 late response hypomethylation
cg01616394	NCOR2 (-86254), SCARB1 (+210255)	12	125138263					CEBPB	1	-0.1072724	-0.1003832	-0.0473392	-0.1472297	-0.0599865	-0.0882214 late response hypomethylation
cg05588496	DOK6 (-562)	18	67067721	DOK6	TSS1500	chr18:67067509-67069168	Island	HA-E2F1|TAF1|Pol2	1	-0.1381109	0.10478305	0.0664623	-0.1478009	-0.0140745	-0.0117939 oscillatory response
cg00421815	ARL15 (+260712), NDUFS4 (+489226)	5	53345690	ARL15	Body			NA	0	-0.1893867	0.02122906	0.03228424	-0.1492792	0.03353931	-0.0293115 transient response hypomethylation
cg18687085	ALPK2 (+228726), NEDD4L (+355853)	18	56067462	NEDD4L	3'UTR			CTCF|SMC3_(ab926:	1	-0.1176289	0.0188821	-0.0760374	-0.1494826	0.08978273	-0.0244504 oscillatory response
cg18424634	ZIC1 (-83)	3	147127097	ZIC1	TSS200	chr3:147126988-147128999	Island	SUZ12|NANOG_(SC-	1	-0.293494	-0.2552738	-0.0719918	-0.1498317	-0.2242581	-0.3079165 constant hypomethylation
cg19329225	CCDC134 (-3957)	22	42192720	MEI1	Body	chr22:42195953-42196961	N_Shelf	NA	0	-0.1566072	0.02468807	0.08440236	-0.1508473	-0.001614	0.00388292 oscillatory response

cg05372444	UPK3B (+5928), POMZP3 (+110947)	7	76145672	UPK3B;UPK3	3'UTR;3'UTR	chr7:76145396-76145781	Island	HEY1	0	-0.1143094	-0.1286186	0.12537956	-0.1534394	-0.0710654	-0.0061759	oscillatory response
cg01799418	NONE	3	117428927					NA	0	-0.1768691	-0.0180526	-0.0700015	-0.1536574	-0.0126785	-0.0157735	early response hypomethylation
cg20931867	PET112 (-2177)	4	152684351			chr4:152682041-152682293	S_Shelf	NA	0	-0.1561998	-0.0339002	-0.0013443	-0.1541588	-0.0207969	-0.0371909	transient response hypomethylation
cg13226172	CNTFR (+353)	9	34589381	CNTFR;CNTFI	5'UTR;5'UTR	chr9:34589113-34591978	Island	NA	0	-0.1254988	0.10436919	0.01987859	-0.1546774	0.00421586	0.01396054	transient response hypomethylation
cg26561082	ZMYND11 (+184430), DIP2C (+325244)	10	410363	DIP2C	Body	chr10:409201-409523	S_Shore	NA	0	-0.1810535	-0.177243	-0.0406948	-0.1549523	-0.234016	-0.2310612	late response hypomethylation
cg18083595	USP29 (-847)	19	57630661	USP29	TSS1500	chr19:57630339-57630633	S_Shore	NA	0	-0.1003153	0.07431412	-0.0471683	-0.155573	0.00642768	-0.0198308	transient response hypomethylation
cg04626565	MAF (+1837)	16	79632784	MAF;MAF	1stExon;1stExon	chr16:79632619-79635537	Island	CTCF|Rad21|CTCF_(	1	-0.1139743	0.01784518	0.00042793	-0.1566539	0.03446431	0.04350472	transient response hypomethylation
cg11512009	THRA (+1627), NR1D1 (+36278)	17	38220694	THRA;THRA	5'UTR;5'UTR	chr17:38219084-38219880	S_Shore	Sin3Ak-20|Max|Pbx	1	-0.1179001	-0.0162332	0.00832866	-0.1623697	-0.0294127	-0.1516232	oscillatory response
cg17657037	ITGB1BP3 (+756)	19	3933856	ITGB1BP3	Body	chr19:3933473-3933816	S_Shore	NA	0	-0.1172539	0.10164032	0.01700443	-0.1642339	-0.0244702	0.01075868	transient response hypomethylation
cg10614045	DBX1 (-253)	11	20182122	DBX1	TSS1500	chr11:20181200-20182325	Island	NA	1	-0.1592359	-0.0151248	0.0469448	-0.1642979	0.0164946	0.02826881	transient response hypomethylation
cg19537969	EPYC (+40893)	12	91357909	EPYC	3'UTR			NA	0	-0.1498271	-0.0059724	0.00709343	-0.1654326	0.10966063	-0.1036467	oscillatory response
cg09540629	UTF1 (-62018), KNDC1 (+7789)	10	134981759	KNDC1	Body	chr10:134980302-134981059	S_Shore	NA	0	-0.1460693	0.06206877	0.0413081	-0.1808512	-0.166216	0.01485811	transient response hypomethylation
cg17983217	DDAH2 (-185)	6	31698223	DDAH2	TSS200	chr6:31695894-31698245	Island	Pol2-4H8|Pol2|TBP|	1	-0.3192856	-0.2173157	-0.203754	-0.1814438	-0.29932	-0.3218273	constant hypomethylation
cg08447739	HTRA1 (-681)	10	124220359	HTRA1	TSS1500	chr10:124220338-124222240	Island	NA	1	-0.2159981	-0.2676722	-0.0368496	-0.1822048	-0.3418058	-0.2887986	late response hypomethylation
cg06053651	DNAJB13 (-995)	11	73660368	DNAJB13	TSS1500			NA	0	-0.1129538	-0.0455695	0.05155178	-0.188301	0.01943193	-0.1404175	oscillatory response
cg03713642	HS3ST2 (-759)	16	22825100	HS3ST2	TSS1500	chr16:22824616-22826459	Island	NA	1	-0.16491	0.0147481	-0.0541004	-0.1956891	-0.0042444	-0.0325446	early response hypomethylation
cg07571282	GAN (-53475), BCMO1 (+22800)	16	81295095	BCMO1	Body			NA	0	-0.1326593	0.05394687	0.06068568	-0.1970266	-0.1139914	-0.069536	oscillatory response
cg19147129	TET3 (-43139), DGUOK (+76358)	2	74230310			chr2:74229477-74229996	S_Shore	NA	1	-0.1469175	0.01064123	0.04519355	-0.1991395	0.0084357	-0.1048115	oscillatory response
cg14173033	WDR45L (+61100), FOXK2 (+67717)	17	80545310	FOXK2	Body	chr17:80545433-80545658	N_Shore	NA	1	-0.1538752	-0.0283978	-0.0250096	-0.2023679	0.02873981	0.04119221	transient response hypomethylation
cg20104640	ABRA (+106179), OXR1 (+216141)	8	107676292	OXR1;OXR1	Body;Body			NA	0	-0.2512685	-0.2648285	-0.1925902	-0.2051379	-0.2604451	-0.3014269	constant hypomethylation
cg00003994	MEOX2 (+445)	7	15725862	MEOX2	1stExon			NA	0	-0.1278661	0.02362277	-0.0952422	-0.2290547	0.08081704	0.04834553	oscillatory response
cg15706807	FAM110B (-800514), IMPAD1 (-200169)	8	58106598					NA	0	-0.3161287	-0.2864641	-0.2903612	-0.2428694	-0.286256	-0.3285706	constant hypomethylation
cg00984474	FASTKD3 (+18227), ADCY2 (+454580)	5	7850922	C5orf49	Body	chr5:7850957-7851413	N_Shore	NA	1	-0.4977016	-0.3669299	-0.484895	-0.3624148	-0.4724401	-0.4898058	constant hypomethylation

Supplementary Table 1. **(B)** Replicated infection induced differentially methylated probes (iDMPs) at T4 in infected versus uninfected human macrophages.

Methylation sites and annotation										Methylation level differences (Δβ)						
Probe	Gene annotation using GREAT	Chromosome (chr)	Position	UCSC REFGENE NAME	UCSC REFGENE GROUP	UCSC CPG ISLANDS NAME	RELATION TO UCSC CPG ISLAND	Transcription Factor Binding Site Annotation	DHS Annotation	Δβ T2 infected versus uninfected Experiment 1	Δβ T4 infected versus uninfected Experiment 1	Δβ T1 infected versus uninfected Experiment 2	Δβ T2 infected versus uninfected Experiment 2	Δβ T3 infected versus uninfected Experiment 2	Δβ T4 infected versus uninfected Experiment 2	Differential methylation pattern
cg27512082	SKA2 (-90)	17	57232889	SKA2;PRR11;SKA2	TSS200;TSS1500;TSS200	chr17:57231855-57232655	S_Shore	HA-E2F1|NF-Y	1	0.32989302	0.27991136	0.0830382	0.08453494	0.20980772	0.2038327	constant hypermethylation
cg24860775	GABRA1 (-1646)	5	161275745	GABRA1	5'UTR			NA	0	-0.1143332	0.12250587	0.05054754	0.07972699	-0.0979078	0.19984782	oscillatory response
cg15718555	TWIST2 (+90825), HDAC4 (+475145)	2	239847497					NA	0	0.00695561	0.15079315	0.00743458	0.06187061	-0.0195494	0.19406809	oscillatory response
cg12816057	ADRB2 (-171949), HTR4 (-17583)	5	148034206	HTR4	TSS1500	chr5:148033472-148034080	S_Shore	PU.1	1	0.10604515	0.11194423	-0.1518773	0.10233968	-0.0232675	0.1668183	oscillatory response
cg16430616	ASAP2 (-200)	2	9346693	ASAP2	TSS200	chr2:9346383-9347944	Island	HA-E2F1|AP-2	1	-0.137199	0.10497647	0.07805858	-0.0027916	-0.0238408	0.15255346	oscillatory response
cg20539449	CDH6 (+167374), DROSHA (+171146)	5	31361135					NA	0	-0.0122368	0.12011439	0.01144819	0.02579371	-0.0613251	0.15127931	oscillatory response
cg04094169	CADM3 (+517)	1	159141893	CADM3	Body	chr1:159141202-159141718	S_Shore	NRSF	1	0.05308585	0.11287346	-0.0936982	-0.0339985	-0.0598621	0.14084701	oscillatory response
cg10979880	BVES (-147)	6	105584689	BVES;BVES	TSS1500;TSS200	chr6:105584148-105585621	Island	CTCF|TBP	1	0.02065229	0.10360739	-0.0061428	0.0164302	-0.0183121	0.13955864	late response hypermethylation
cg03613077	RGS7 (-427)	1	241520904	RGS7	TSS1500	chr1:241520103-241520790	S_Shore	NA	0	-0.0251415	0.12305581	-0.0903533	-0.0367746	0.02786859	0.13449663	oscillatory response
cg22490991	UNC5C (+4858), BMPR1B (+786375)	4	96465502	UNC5C	Body	chr4:96469456-96469667	N_Shelf	NA	0	-0.0111646	0.1010858	0.0501672	0.01570504	-0.0783523	0.13401105	oscillatory response
cg26313152	PNPLA6 (-797)	19	7598793	PNPLA6;MCOLN1	TSS1500;3'UTR	chr19:7598413-7601008	Island	NA	0	0.11421283	0.13131061	0.0940138	-0.0686498	-0.0454505	0.12635574	oscillatory response
cg03972398	HOXC13 (+604)	12	54333179	HOXC13	1stExon	chr12:54332805-54333731	Island	NA	1	-0.1255517	0.11864824	-0.0589809	-0.1060001	0.04847834	0.12587368	oscillatory response
cg26539042	ENGASE (-41710), C1QTNF1 (+9058)	17	77029308	C1QTNF1;C1QTNF1;C1QTNF1	5'UTR;TSS1500;5'UTR			USF2|USF-1	1	-0.0044153	0.13850829	-0.1253363	-0.1005104	-0.0694942	0.12324242	oscillatory response
cg19721115	KCNMB4 (-396)	12	70759665	KCNMB4	TSS1500	chr12:70759437-70761052	Island	AP-2alpha|AP-	1	-0.0803165	0.12491303	0.00704675	0.11079362	-0.0094136	0.12081086	oscillatory response
cg05022301	C14orf109 (-859), MOAP1 (+733)	14	93650515	C14orf109;MOAP1;C14orf109	TSS1500;Body;TSS1500	chr14:93650745-93651652	N_Shore	NA	0	0.03308241	0.10725109	0.02542236	0.02228323	0.09875105	0.12059459	late response hypermethylation
cg10870083	LPHN3 (-294653)	4	62068185			chr4:62065883-62068801	Island	NA	0	0.01818395	0.20950513	-0.0516095	0.06964951	0.03649045	0.12026422	oscillatory response
cg03375453	TBCEL (-177)	11	120894635	TBCEL	TSS200	chr11:120894689-120895082	N_Shore	ZNF263|EBF|S	1	-0.0247489	0.1653192	-0.0728127	0.12772813	-0.020762	0.11512583	oscillatory response
cg18949192	TBX3 (-12312)	12	115134280			chr12:115135925-115136350	N_Shore	SUZ12	0	0.03558175	0.13172402	-0.006839	0.01588017	-0.075864	0.11310292	oscillatory response
cg10868030	SDHA (-25705), PLEKHG4B (+52278)	5	192650	LRRC14B	Body	chr5:191792-192544	S_Shore	NA	0	0.00458726	0.10130227	-0.0172024	-0.0394961	-0.0564491	0.11179775	oscillatory response
cg06284169	PAK2 (-250)	3	196466477	PAK2	TSS1500	chr3:196466585-196467353	N_Shore	HA-E2F1	0	-0.0178399	0.21449352	-0.0165399	-0.0029459	0.05438358	0.10911325	late response hypermethylation
cg21823425	TMEM225 (-992)	11	123757331	TMEM225	TSS1500			NA	0	0.09451731	0.12698698	0.06883531	0.0666017	0.06886124	0.10859409	constant hypermethylation
cg14041283	ADAMTSL1 (-567740), SH3GL2 (+327386)	9	17906338			chr9:17906419-17907488	N_Shore	SUZ12	0	-0.0710618	0.13827693	0.03122934	-0.0421058	0.03900526	0.10672337	late response hypermethylation
cg23208176	IRX4 (+6711), NDUFS6 (+74673)	5	1876168			chr5:1874907-1879032	Island	SUZ12	0	-0.0822624	0.13215543	-0.1015533	0.0608113	0.04036602	0.10602995	oscillatory response
cg05225993	EZH2 (+43537), CUL1 (+141971)	7	148537903	EZH2;EZH2	Body;Body			NA	0	-0.1113832	0.11693716	0.04819921	0.01202063	-0.0602311	0.10584812	oscillatory response
cg22396878	MAB21L2 (-405877), DCLK2 (+97774)	4	151097199	DCLK2	Body			NA	1	0.04303719	0.23458027	0.17304904	-0.0346722	-0.0165556	0.10407058	oscillatory response
cg23056703	EDEM1 (+241430)	3	5470788					Pol2|GATA-1|S	1	0.03498786	0.10866992	-0.0400255	0.04595075	0.02266736	0.10382511	late response hypermethylation
cg17459298	ADAMTS20 (-618)	12	43946341	ADAMTS20	TSS1500	chr12:43944719-43946285	S_Shore	NA	0	0.07491947	0.13151877	-0.0103565	-0.0288219	0.03148102	0.10306119	late response hypermethylation
cg05173737	SYT16 (+121465), KCNH5 (+927949)	14	62584005	FLJ43390	TSS200	chr14:62583679-62584279	Island	Max|USF-1|Ju	1	-0.044541	0.12287126	-0.0057436	0.02983678	0.01959259	0.10250323	late response hypermethylation
cg04124626	PLCXD3 (+795)	5	41509934	PLCXD3	Body	chr5:41509783-41510166	Island	NA	0	0.17758974	0.15359533	0.01740987	0.04872579	0.05140834	0.10229097	late response hypermethylation
cg00332146	CDHR2 (-441)	5	175975901	PCDH24	TSS1500			NA	1	-0.094941	0.13078127	-0.0147358	0.00275407	-0.0345572	0.10150079	late response hypermethylation
cg26673279	ROBO3 (+4127), ROBO4 (+28399)	11	124739431	ROBO3	Body	chr11:124738712-124739011	S_Shore	NA	0	0.0451294	0.10920935	-0.0266471	-0.0294009	-0.0269588	0.10134066	late response hypermethylation
cg20729747	HIST4H4 (-4019), H2AFJ (+814)	12	14928083	H2AFJ;H2AFJ;H2AFJ	1stExon;Body;3'UTR	chr12:14927291-14928023	S_Shore	Pol2	1	-0.0447627	-0.1256084	-0.0715742	-0.0842464	0.01041293	-0.1000106	oscillatory response
cg13309828	PCDHAC2 (-122482), ZMAT2 (+143233)	5	140223264	PCDHA6;PCDHA2;PCDHA1	Body;Body;Body	chr5:140221007-140221381	S_Shore	NA	0	-0.0222658	-0.1192985	0.07000103	0.02313032	0.04215879	-0.1001241	oscillatory response
cg11666343	OTOS (-185881), GPC1 (-109161)	2	241265953			chr2:241262311-241262759	S_Shelf	NA	0	-0.0002437	-0.1122012	-0.0318608	0.03129484	0.04403248	-0.1001553	late response hypomethylation
cg13120108	HIF1A (-372081), PRKCH (+1523)	14	61790037	PRKCH	Body	chr14:61787880-61789467	S_Shore	EBF	1	0.01568219	-0.1312863	0.08258152	0.0354496	-0.3465488	-0.1003453	oscillatory response
cg13073030	BCL11B (-28480), SETD3 (+180924)	14	99766301					NA	0	0.04448395	-0.1343296	0.01818454	0.0513997	0.11929613	-0.1003821	oscillatory response
cg24631834	NKX6-1 (+213675), AGPAT9 (+748055)	4	85205711					NA	0	0.01247008	-0.1074421	0.13111821	-0.0001355	-0.1122748	-0.1007978	oscillatory response
cg13683218	COL24A1 (-399)	1	86622552	COL24A1	TSS1500	chr1:86621278-86622871	Island	ZNF263|KAP1|	1	-0.1730739	-0.1341252	-0.0057613	0.00562983	0.03092346	-0.1008198	late response hypomethylation
cg25889998	TUSC5 (+31995), YWHAE (+88604)	17	1214951					NA	0	0.18149121	-0.1400181	-0.0102112	-0.0725172	-0.0156183	-0.10091	oscillatory response
cg18465515	HIST1H3G (-1571), HIST1H2BI (-21)	6	26273182	HIST1H2BI	TSS200	chr6:26273290-26273557	N_Shore	Pol2|TBP|RFX5	1	0.01329728	-0.1589962	-0.0543427	-0.0293085	-0.0361021	-0.1014236	oscillatory response
cg16333262	ESRRB (-219934), IFT43 (+165660)	14	76617755	C14orf118	TSS1500	chr14:76618138-76618452	N_Shore	NA	0	0.02372747	-0.1067817	-0.011376	-0.0766099	0.12890216	-0.1017792	oscillatory response
cg25304681	RPTN (-889)	1	152132592	RPTN	TSS1500			NA	0	0.03251221	-0.1496423	0.11332461	0.14029696	-0.0192584	-0.1019798	oscillatory response
cg03995567	KCTD17 (+427)	22	37448205	KCTD17	Body	chr22:37447689-37448238	Island	YY1_(C-20)|YY	1	-0.0718753	-0.1526559	0.02179603	0.04467105	-0.0625466	-0.1020888	late response hypomethylation
cg20396423	EPN2 (-36)	17	19140653	EPN2	TSS200	chr17:19140307-19141648	Island	Egr-1|Pol2-4H8	1	-0.1794208	-0.2146498	-0.0347484	-0.0491212	-0.0919107	-0.1022673	late response hypomethylation
cg22811233	GREB1 (-50955), E2F6 (-16990)	2	11623286			chr2:11622000-11623297	Island	HA-E2F1|Pol2|	1	0.19118591	-0.2049233	0.00622291	-0.001075	0.0790094	-0.1025275	oscillatory response
cg13916967	SOX6 (-210692), C11orf58 (-125043)	11	16635104			chr11:16634957-16635969	Island	SUZ12	0	0.01726811	-0.116435	0.03058839	0.01632447	-0.027021	-0.1032956	late response hypomethylation
cg11374425	FEZF2 (-488)	3	62359677	FEZF2	TSS1500	chr3:62357639-62359774	Island	SUZ12	0	-0.0677775	-0.1164732	-0.1457137	-0.0425342	-0.027963	-0.1034172	oscillatory response
cg05186793	KIAA1267 (+34751), STH (+158905)	17	44235520	KIAA1267	Body			NA	0	0.0460623	-0.1097148	0.14861893	-0.0184216	0.01658084	-0.1040174	oscillatory response
cg13204325	RIMBP2 (-165125), STX2 (+156224)	12	131167586			chr12:131171168-131171548	N_Shelf	NA	0	0.06947477	-0.110212	-0.0440245	0.14323967	0.03241389	-0.1044241	oscillatory response
cg26685539	VRK1 (+3443)	14	97267126	VRK1	5'UTR	chr14:97263559-97264274	S_Shelf	NA	0	-0.0085972	-0.1134319	0.02662419	0.13311444	0.110655	-0.1045305	oscillatory response
cg21654379	CCR4 (-128758), TRIM71 (+4798)	3	32864307	TRIM71	Body	chr3:32863174-32863415	S_Shore	NA	0	0.04142464	-0.1245782	0.04570289	0.05432832	0.01360988	-0.1052778	oscillatory response
cg23479949	GABRP (-281162), KCNMB1 (-112923)	5	169929560	KCNIP1;KCNIP1	TSS1500;Body	chr5:169931065-169931471	N_Shore	NA	0	0.00549028	-0.1422166	0.00538794	-0.0423829	-0.003156	-0.1055526	late response hypomethylation
cg20500869	NPTX1 (+27919), ENDOV (+33518)	17	78422484					NA	0	0.0432311	-0.1056895	0.02715041	0.00023643	-0.0448621	-0.1056396	late response hypomethylation
cg09554951	CDKN1C (+16269), KCNQ1 (+424505)	11	2890725	KCNQ1DN	TSS1500	chr11:2890388-2891337	Island	NA	0	-0.1815007	-0.1494997	-0.0295571	-0.0447866	-0.104116	-0.1057251	late response hypomethylation
cg19774868	ADNP2 (+38494), PARD6G (+99988)	18	77905408	LOC100130522	TSS1500	chr18:77905297-77905566	Island	NA	0	-0.1435119	-0.1890046	-0.0092896	-0.0689802	-0.1240448	-0.1063192	late response hypomethylation
cg14113958	TNFSF15 (-88830), TNFSF8 (+35637)	9	117657237					FOXA1_(SC-10	1	0.0052998	-0.1079843	-0.0101066	-0.0363476	-0.0462902	-0.1064643	late response hypomethylation
cg27244120	IL34 (-3363)	16	70610434	SF3B3	3'UTR			IRF4_(M-17)|M	1	-0.0071005	-0.1387047	0.02326556	-0.0062346	-0.0041091	-0.1066655	late response hypomethylation

<table>
<tr><td>cg04362790</td><td>EML1 (-35933), CYP46A1 (+73057)</td><td>14</td><td>100223811</td><td></td><td></td><td></td><td></td><td>Egr-1</td><td>0</td><td>0.10115596</td><td>-0.1180262</td><td>0.01354834</td><td>0.06501801</td><td>0.01119541</td><td>-0.1076836</td><td>oscillatory response</td></tr>
<tr><td>cg07569293</td><td>RNF169 (+91245), XRRA1 (+109074)</td><td>11</td><td>74551157</td><td>RNF169</td><td>3'UTR</td><td></td><td></td><td>NA</td><td>1</td><td>-0.0246231</td><td>-0.1318684</td><td>0.14388242</td><td>0.08366105</td><td>0.02518154</td><td>-0.1078571</td><td>oscillatory response</td></tr>
<tr><td>cg02221053</td><td>HACE1 (-84064), LIN28B (-13065)</td><td>6</td><td>105391857</td><td></td><td></td><td>chr6:105388190-105389545</td><td>S_Shelf</td><td>NRSF|YY1_(C-2</td><td>1</td><td>0.03659399</td><td>-0.1085076</td><td>0.02440054</td><td>0.05146037</td><td>0.11736485</td><td>-0.1089479</td><td>oscillatory response</td></tr>
<tr><td>cg12183978</td><td>PAM (+1545), GIN1 (+252770)</td><td>5</td><td>102203071</td><td>PAM</td><td>Body</td><td>chr5:102201583-102201912</td><td>S_Shore</td><td>NA</td><td>0</td><td>-0.0335676</td><td>-0.1028201</td><td>0.04051879</td><td>0.01277053</td><td>-0.0301415</td><td>-0.1096446</td><td>late response hypomethylation</td></tr>
<tr><td>cg22500102</td><td>YOD1 (-2409), PFKFB2 (+211)</td><td>1</td><td>207226830</td><td>PFKFB2</td><td>5'UTR</td><td>chr1:207226281-207226842</td><td>Island</td><td>Pol2|CTCF|Pol</td><td>1</td><td>-0.0813926</td><td>-0.1989596</td><td>-0.0605754</td><td>0.04878418</td><td>-0.1444618</td><td>-0.1100395</td><td>oscillatory response</td></tr>
<tr><td>cg22365990</td><td>TNS3 (-159103), C7orf69 (-54044)</td><td>7</td><td>47780844</td><td></td><td></td><td></td><td></td><td>NA</td><td>0</td><td>-0.1654842</td><td>-0.1157554</td><td>0.04633608</td><td>0.03845863</td><td>0.02320469</td><td>-0.1100483</td><td>late response hypomethylation</td></tr>
<tr><td>cg07573715</td><td>ADCK3 (+36059), CDC42BPA (+341829)</td><td>1</td><td>227163996</td><td>CABC1</td><td>Body</td><td>chr1:227163739-227164237</td><td>Island</td><td>NA</td><td>0</td><td>-0.2444129</td><td>-0.2436541</td><td>-0.0439382</td><td>-0.0349211</td><td>-0.1587447</td><td>-0.1102607</td><td>late response hypomethylation</td></tr>
<tr><td>cg08376864</td><td>HRASLS (-191)</td><td>3</td><td>192958726</td><td>MGC2889;HRASLS</td><td>TSS1500;TSS200</td><td>chr3:192958709-192959373</td><td>Island</td><td>GATA3_(SC-26</td><td>1</td><td>-0.0062605</td><td>-0.2143063</td><td>0.01849162</td><td>-0.1436633</td><td>-0.01709</td><td>-0.1107582</td><td>oscillatory response</td></tr>
<tr><td>cg19611602</td><td>CNTN3 (-924)</td><td>3</td><td>74571266</td><td>CNTN3</td><td>TSS1500</td><td></td><td></td><td>NA</td><td>0</td><td>0.04670891</td><td>-0.1016581</td><td>0.0261728</td><td>-0.0528309</td><td>0.06906263</td><td>-0.1113263</td><td>oscillatory response</td></tr>
<tr><td>cg19791043</td><td>SSTR5 (-6394), SOX8 (+84554)</td><td>16</td><td>1116361</td><td>LOC146336</td><td>Body</td><td>chr16:1115827-1116468</td><td>Island</td><td>NA</td><td>0</td><td>0.04744962</td><td>-0.1180975</td><td>-0.0063323</td><td>0.01315691</td><td>0.00184526</td><td>-0.1123837</td><td>late response hypomethylation</td></tr>
<tr><td>cg06984764</td><td>TARS (-142788), NPR3 (+586672)</td><td>5</td><td>33298109</td><td></td><td></td><td></td><td></td><td>GABP|ELF1_(S</td><td>0</td><td>-0.3210767</td><td>-0.2732599</td><td>-0.0578957</td><td>-0.0706398</td><td>-0.1396334</td><td>-0.1128106</td><td>constant hypomethylation</td></tr>
<tr><td>cg03127886</td><td>FOXF1 (-577895), IRF8 (+33464)</td><td>16</td><td>85966237</td><td></td><td></td><td></td><td></td><td>NA</td><td>0</td><td>-0.186552</td><td>-0.114993</td><td>-0.109293</td><td>-0.097904</td><td>-0.1440655</td><td>-0.1130313</td><td>constant hypomethylation</td></tr>
<tr><td>cg10238145</td><td>ACTR3 (-3329)</td><td>2</td><td>114644207</td><td></td><td></td><td>chr2:114647669-114648312</td><td>N_Shelf</td><td>c-Fos|YY1_(C-2</td><td>1</td><td>0.04049655</td><td>-0.1205968</td><td>-0.103205</td><td>-0.0621912</td><td>0.13804811</td><td>-0.1131194</td><td>oscillatory response</td></tr>
<tr><td>cg03586128</td><td>TUBGCP3 (-57316), ATP11A (-44846)</td><td>13</td><td>113299796</td><td></td><td></td><td>chr13:113303306-113303540</td><td>N_Shelf</td><td>NA</td><td>0</td><td>-0.0836847</td><td>-0.1233373</td><td>-0.0211914</td><td>-0.0135875</td><td>-0.0256978</td><td>-0.1139188</td><td>late response hypomethylation</td></tr>
<tr><td>cg18450625</td><td>EPB42 (-1305)</td><td>15</td><td>43514627</td><td>EPB42</td><td>TSS1500</td><td></td><td></td><td>NA</td><td>0</td><td>0.01760372</td><td>-0.1047247</td><td>-0.0129898</td><td>-0.1000302</td><td>0.10513364</td><td>-0.1140174</td><td>oscillatory response</td></tr>
<tr><td>cg10478310</td><td>PKD2 (+860)</td><td>4</td><td>88929658</td><td>PKD2</td><td>Body</td><td>chr4:88928480-88929659</td><td>Island</td><td>HA-E2F1|Pol2|</td><td>1</td><td>0.06204132</td><td>-0.126685</td><td>0.03222369</td><td>0.08003299</td><td>-0.0014185</td><td>-0.1142775</td><td>oscillatory response</td></tr>
<tr><td>cg26783481</td><td>POU5F2 (-48587), FAM172A (+321508)</td><td>5</td><td>93125895</td><td>FAM172A</td><td>Body</td><td></td><td></td><td>NA</td><td>0</td><td>0.09488132</td><td>-0.1364448</td><td>0.09785802</td><td>-0.0301792</td><td>0.00695067</td><td>-0.1143008</td><td>oscillatory response</td></tr>
<tr><td>cg03983008</td><td>RUFY1 (+24698), HNRNPH1 (+48453)</td><td>5</td><td>179002268</td><td>RUFY1</td><td>Body</td><td>chr5:179004497-179004836</td><td>N_Shelf</td><td>NA</td><td>0</td><td>0.07509009</td><td>-0.1112078</td><td>0.06379065</td><td>0.1052807</td><td>0.04279338</td><td>-0.114584</td><td>oscillatory response</td></tr>
<tr><td>cg19636308</td><td>PLD6 (-15054), FLCN (+15802)</td><td>17</td><td>17124699</td><td>FLCN</td><td>Body</td><td>chr17:17124698-17124931</td><td>Island</td><td>NA</td><td>0</td><td>-0.1865482</td><td>-0.1273585</td><td>-0.0547827</td><td>-0.04999</td><td>-0.0876869</td><td>-0.1147569</td><td>oscillatory response</td></tr>
<tr><td>cg12615761</td><td>RAB12 (-535)</td><td>18</td><td>8608907</td><td>RAB12</td><td>TSS1500</td><td>chr18:8608763-8610370</td><td>Island</td><td>ELF1_(SC-631)</td><td>1</td><td>-0.0844456</td><td>-0.151748</td><td>-0.0141811</td><td>-0.0903529</td><td>-0.1111197</td><td>-0.114928</td><td>late response hypomethylation</td></tr>
<tr><td>cg26858540</td><td>ZNF787 (+29326), NLRP5 (+92231)</td><td>19</td><td>56603322</td><td>ZNF787</td><td>Body</td><td>chr19:56598038-56600296</td><td>S_Shelf</td><td>IRF4_(M-17)</td><td>1</td><td>-0.3781738</td><td>-0.204009</td><td>-0.0425342</td><td>-0.0578739</td><td>-0.1076638</td><td>-0.1150161</td><td>late response hypomethylation</td></tr>
<tr><td>cg10850725</td><td>ZNF233 (+348)</td><td>19</td><td>44764380</td><td>ZNF233</td><td>5'UTR</td><td>chr19:44763978-44764312</td><td>S_Shore</td><td>NA</td><td>0</td><td>0.10166674</td><td>-0.1263727</td><td>0.0704009</td><td>-0.0590796</td><td>-0.001954</td><td>-0.1170597</td><td>oscillatory response</td></tr>
<tr><td>cg22694703</td><td>TEAD1 (-64)</td><td>11</td><td>12695904</td><td>TEAD1</td><td>TSS200</td><td>chr11:12695414-12696981</td><td>Island</td><td>Pol2|HA-E2F1|</td><td>1</td><td>-0.1032365</td><td>-0.1194659</td><td>0.06139764</td><td>-0.0566004</td><td>-0.0552646</td><td>-0.1173826</td><td>oscillatory response</td></tr>
<tr><td>cg15618525</td><td>SCGB3A1 (-4423)</td><td>5</td><td>180022909</td><td></td><td></td><td>chr5:180017099-180019062</td><td>S_Shelf</td><td>HA-E2F1|GAT</td><td>0</td><td>-0.1659012</td><td>-0.1076548</td><td>-0.008515</td><td>-0.0067576</td><td>-0.1502243</td><td>-0.1181932</td><td>late response hypomethylation</td></tr>
<tr><td>cg13206017</td><td>SST (-25)</td><td>3</td><td>187388225</td><td>SST</td><td>TSS200</td><td>chr3:187387914-187388176</td><td>S_Shore</td><td>TBP</td><td>1</td><td>0.04396687</td><td>-0.1294316</td><td>0.06197469</td><td>0.01424141</td><td>0.00505073</td><td>-0.1184144</td><td>oscillatory response</td></tr>
<tr><td>cg14585163</td><td>ZNRD1 (-42014), HLA-J (+12805)</td><td>6</td><td>29987021</td><td>NCRNA00171</td><td>Body</td><td></td><td></td><td>NA</td><td>0</td><td>-0.1699703</td><td>-0.110858</td><td>-0.0077639</td><td>-0.0238236</td><td>-0.1166978</td><td>-0.1193925</td><td>late response hypomethylation</td></tr>
<tr><td>cg26898932</td><td>NSRP1 (-1353)</td><td>17</td><td>28442480</td><td>CCDC55</td><td>TSS1500</td><td>chr17:28443746-28444011</td><td>N_Shore</td><td>STAT3|p300_(</td><td>1</td><td>-0.0984167</td><td>-0.1325849</td><td>-0.0255716</td><td>0.0584006</td><td>0.06390013</td><td>-0.1196321</td><td>oscillatory response</td></tr>
<tr><td>cg19109130</td><td>PTDSS1 (-4017), MTERFD1 (+3646)</td><td>8</td><td>97270149</td><td>MTERFD1</td><td>Body</td><td>chr8:97273615-97274767</td><td>N_Shelf</td><td>NA</td><td>0</td><td>-0.0046094</td><td>-0.1084342</td><td>0.07054529</td><td>0.07229895</td><td>-0.0183321</td><td>-0.1197364</td><td>oscillatory response</td></tr>
<tr><td>cg27621528</td><td>GPC1 (+17503), ANKMY1 (+104787)</td><td>2</td><td>241392617</td><td>PP14571;GPC1</td><td>Body;Body</td><td>chr2:241391876-241393598</td><td>Island</td><td>Pol2</td><td>0</td><td>0.11781393</td><td>-0.1058362</td><td>0.19486726</td><td>0.17622012</td><td>-0.1305379</td><td>-0.1199211</td><td>oscillatory response</td></tr>
<tr><td>cg27312944</td><td>SKI (-34157), PRKCZ (+144068)</td><td>1</td><td>2125976</td><td>C1orf86;LOC100128003;C1orf86</td><td>Body;TSS1500;Body</td><td>chr1:2126048-2126642</td><td>N_Shore</td><td>E2F6_(H-50)</td><td>0</td><td>-0.0150799</td><td>-0.1335762</td><td>0.02482391</td><td>0.09171004</td><td>0.11079492</td><td>-0.1212062</td><td>oscillatory response</td></tr>
<tr><td>cg22295435</td><td>VSTM2A (+5846), SEC61G (+211074)</td><td>7</td><td>54615864</td><td>VSTM2A</td><td>Body</td><td>chr7:54614052-54614797</td><td>S_Shore</td><td>NA</td><td>0</td><td>-0.2048787</td><td>-0.1467435</td><td>-0.0912652</td><td>-0.0581811</td><td>-0.0341136</td><td>-0.1219017</td><td>oscillatory response</td></tr>
<tr><td>cg19021466</td><td>SLC6A15 (+863)</td><td>12</td><td>85305742</td><td>SLC6A15</td><td>5'UTR</td><td>chr12:85305663-85305876</td><td>Island</td><td>ZNF263|Max|</td><td>1</td><td>0.07618606</td><td>-0.1361491</td><td>0.02285664</td><td>-0.020195</td><td>0.02828884</td><td>-0.1226579</td><td>late response hypomethylation</td></tr>
<tr><td>cg21745586</td><td>MANSC1 (-15707), DUSP16 (+196572)</td><td>12</td><td>12518875</td><td>LOH12CR1</td><td>Body</td><td></td><td></td><td>USF1_(SC-898</td><td>0</td><td>0.00175285</td><td>-0.1289196</td><td>0.02641217</td><td>-0.1356141</td><td>-0.0775041</td><td>-0.1231095</td><td>late response hypomethylation</td></tr>
<tr><td>cg17280299</td><td>CCNI2 (-446)</td><td>5</td><td>132082690</td><td>CCNI2</td><td>TSS1500</td><td>chr5:132082873-132083911</td><td>N_Shore</td><td>CTCF|Rad21|C</td><td>1</td><td>-0.0677122</td><td>-0.135692</td><td>-0.0120074</td><td>-0.0094975</td><td>-0.0762628</td><td>-0.1233526</td><td>late response hypomethylation</td></tr>
<tr><td>cg02432860</td><td>GAB4 (-76)</td><td>22</td><td>17489187</td><td>GAB4</td><td>TSS200</td><td>chr22:17488274-17489194</td><td>Island</td><td>NA</td><td>0</td><td>0.02895962</td><td>-0.1198431</td><td>-0.0015226</td><td>-0.0569297</td><td>0.00871933</td><td>-0.1251223</td><td>oscillatory response</td></tr>
<tr><td>cg10439246</td><td>TBX18 (-350298), NT5E (-335105)</td><td>6</td><td>85824196</td><td></td><td></td><td></td><td></td><td>NA</td><td>1</td><td>-0.0934705</td><td>-0.1203486</td><td>-0.008428</td><td>-0.020145</td><td>0.03766664</td><td>-0.1255889</td><td>late response hypomethylation</td></tr>
<tr><td>cg04926361</td><td>MBIP (-192566), NKX2-1 (+6982)</td><td>14</td><td>36982447</td><td>SFTA3;SFTA3</td><td>5'UTR;1stExon</td><td>chr14:36983440-36983738</td><td>N_Shore</td><td>NA</td><td>1</td><td>-0.1942966</td><td>-0.1464533</td><td>-0.013938</td><td>-0.022406</td><td>-0.088101</td><td>-0.1281301</td><td>late response hypomethylation</td></tr>
<tr><td>cg12491169</td><td>LRRC4C (+177708)</td><td>11</td><td>40137955</td><td>LRRC4C</td><td>5'UTR</td><td></td><td></td><td>NA</td><td>0</td><td>-0.0003273</td><td>-0.1968097</td><td>0.04272637</td><td>-0.042913</td><td>-0.0160994</td><td>-0.1286975</td><td>late response hypomethylation</td></tr>
<tr><td>cg18011045</td><td>PMP22 (-178)</td><td>17</td><td>15164270</td><td>PMP22;PMP22</td><td>5'UTR;TSS200</td><td>chr17:15163930-15164810</td><td>Island</td><td>Pol2|GATA-1|</td><td>1</td><td>-0.1198392</td><td>-0.1060829</td><td>-0.0879511</td><td>-0.0448125</td><td>-0.1388212</td><td>-0.1293407</td><td>oscillatory response</td></tr>
<tr><td>cg22741272</td><td>DAGLB (+26676), RAC1 (+46841)</td><td>7</td><td>6460966</td><td>DAGLB;DAGLB</td><td>Body;Body</td><td></td><td></td><td>NA</td><td>0</td><td>-0.1468607</td><td>-0.100718</td><td>-0.0399707</td><td>-0.0571292</td><td>-0.1342797</td><td>-0.1349642</td><td>late response hypomethylation</td></tr>
<tr><td>cg05088386</td><td>ELF2 (-124)</td><td>4</td><td>140060774</td><td>ELF2</td><td>TSS200</td><td></td><td></td><td>NA</td><td>0</td><td>-0.08074</td><td>-0.1011501</td><td>0.11091648</td><td>0.05542187</td><td>-0.1704443</td><td>-0.1386485</td><td>oscillatory response</td></tr>
<tr><td>cg08819022</td><td>PEX26 (-29238), MICAL3 (-24123)</td><td>22</td><td>18531447</td><td></td><td></td><td>chr22:18531215-18531504</td><td>Island</td><td>NF-YB|USF-1|</td><td>1</td><td>-0.2801206</td><td>-0.2125714</td><td>-0.0468526</td><td>-0.0759724</td><td>-0.1201606</td><td>-0.138738</td><td>late response hypomethylation</td></tr>
<tr><td>cg09096476</td><td>GTF3C6 (-72984), AMD1 (+10792)</td><td>6</td><td>111206778</td><td>AMD1;AMD1</td><td>5'UTR;Body</td><td></td><td></td><td>NA</td><td>0</td><td>0.06225802</td><td>-0.1363428</td><td>-0.0388664</td><td>-0.0007714</td><td>-0.0221971</td><td>-0.1406963</td><td>late response hypomethylation</td></tr>
<tr><td>cg10917941</td><td>TRIM39-RPP21 (-1038)</td><td>6</td><td>30296049</td><td>HCG18;TRIM39;HCG18</td><td>TSS1500;5'UTR;TSS1500</td><td>chr6:30294169-30295071</td><td>S_Shore</td><td>MafK_(ab5032</td><td>0</td><td>-0.0004491</td><td>-0.1003871</td><td>-0.1086924</td><td>-0.0380345</td><td>-0.0266248</td><td>-0.1408524</td><td>oscillatory response</td></tr>
<tr><td>cg02672530</td><td>SCAND1 (-138417), EPB41L1 (-61697)</td><td>20</td><td>34680964</td><td></td><td></td><td>chr20:34680003-34681001</td><td>Island</td><td>Pol2|Ini1</td><td>1</td><td>-0.0411573</td><td>-0.1604691</td><td>-0.0142472</td><td>0.04446463</td><td>-0.0112538</td><td>-0.1417682</td><td>late response hypomethylation</td></tr>
<tr><td>cg05937055</td><td>CACNA1E (-323921), MR1 (+126204)</td><td>1</td><td>181128764</td><td></td><td></td><td></td><td></td><td>GATA-2|c-Fos|</td><td>1</td><td>-0.2074901</td><td>-0.1918941</td><td>-0.0348058</td><td>-0.0528785</td><td>-0.0886611</td><td>-0.1421114</td><td>late response hypomethylation</td></tr>
<tr><td>cg09007434</td><td>MITF (+571987), FOXP1 (+933743)</td><td>3</td><td>70360572</td><td></td><td></td><td></td><td></td><td>NA</td><td>0</td><td>0.06575414</td><td>-0.1051787</td><td>-0.0089881</td><td>-0.0231021</td><td>0.03512477</td><td>-0.1431679</td><td>late response hypomethylation</td></tr>
<tr><td>cg14578009</td><td>OR2V2 (+15700), TRIM7 (+34534)</td><td>5</td><td>180597642</td><td></td><td></td><td>chr5:180596863-180597485</td><td>S_Shore</td><td>NA</td><td>1</td><td>-0.3059022</td><td>-0.2266211</td><td>-0.062427</td><td>0.01626703</td><td>-0.0965381</td><td>-0.1440902</td><td>oscillatory response</td></tr>
<tr><td>cg26985354</td><td>ZNF296 (+12507), CLASRP (+24883)</td><td>19</td><td>45567180</td><td>SFRS16</td><td>Body</td><td>chr19:45567150-45567940</td><td>Island</td><td>NA</td><td>0</td><td>-0.2129355</td><td>-0.1780974</td><td>-0.0608952</td><td>-0.0943765</td><td>-0.2026409</td><td>-0.1447599</td><td>constant hypomethylation</td></tr>
<tr><td>cg06039392</td><td>MTNR1A (-417)</td><td>4</td><td>187476953</td><td>MTNR1A</td><td>TSS1500</td><td>chr4:187476279-187476844</td><td>S_Shore</td><td>NA</td><td>0</td><td>-0.0493133</td><td>-0.1077279</td><td>-0.0546766</td><td>-0.0396987</td><td>-0.0651178</td><td>-0.1478482</td><td>oscillatory response</td></tr>
<tr><td>cg18514949</td><td>SKP1 (-50493), CDKL3 (+139548)</td><td>5</td><td>133563216</td><td>PPP2CA</td><td>TSS1500</td><td>chr5:133561140-133562544</td><td>S_Shore</td><td>Pol2|TBP|TAF</td><td>1</td><td>-0.2537093</td><td>-0.1213724</td><td>0.01935317</td><td>0.0279359</td><td>-0.1358775</td><td>-0.1511803</td><td>late response hypomethylation</td></tr>
<tr><td>cg15150463</td><td>GJA10 (-5098), CASP8AP2 (+59471)</td><td>6</td><td>90599089</td><td></td><td></td><td>chr6:90597207-90597653</td><td>S_Shore</td><td>NA</td><td>0</td><td>-0.0594427</td><td>-0.1003952</td><td>-0.0094866</td><td>-0.0677352</td><td>0.08740388</td><td>-0.1536985</td><td>oscillatory response</td></tr>
<tr><td>cg26842303</td><td>PGCP (-92871), SDC2 (+58746)</td><td>8</td><td>97564627</td><td>SDC2</td><td>Body</td><td></td><td></td><td>NA</td><td>0</td><td>-0.0869906</td><td>-0.1311158</td><td>-0.0411491</td><td>0.03144372</td><td>-0.0703086</td><td>-0.1540918</td><td>late response hypomethylation</td></tr>
<tr><td>cg11988733</td><td>HOXD10 (-12677), HOXD11 (-3269)</td><td>2</td><td>176968814</td><td></td><td></td><td>chr2:176969217-176969895</td><td>N_Shore</td><td>NA</td><td>0</td><td>-0.3419529</td><td>-0.2629349</td><td>-0.0383864</td><td>-0.0130907</td><td>-0.2386142</td><td>-0.156553</td><td>late response hypomethylation</td></tr>
<tr><td>cg22523852</td><td>GABRA5 (-223)</td><td>15</td><td>27112049</td><td>GABRA5;GABRA5;GABRA5</td><td>5'UTR;TSS1500;1stExon</td><td>chr15:27112030-27113479</td><td>Island</td><td>CTCF|Rad21</td><td>1</td><td>-0.1930889</td><td>-0.1280619</td><td>-0.0325953</td><td>-0.0525673</td><td>-0.0903215</td><td>-0.1572671</td><td>late response hypomethylation</td></tr>
<tr><td>cg17159093</td><td>SPATS2L (-100)</td><td>2</td><td>201171255</td><td>SPATS2L;SPATS2L;SPATS2L</td><td>5'UTR;1stExon;TSS200</td><td>chr2:201171200-201172492</td><td>Island</td><td>Pol2|SP1|HEY</td><td>1</td><td>0.04398167</td><td>-0.102282</td><td>-0.039021</td><td>0.14080055</td><td>-0.133384</td><td>-0.1600403</td><td>oscillatory response</td></tr>
<tr><td>cg00962707</td><td>FOXL1 (+103440), FBXO31 (+701839)</td><td>16</td><td>86715554</td><td></td><td></td><td>chr16:86714998-86715325</td><td>S_Shore</td><td>NA</td><td>0</td><td>-0.2075979</td><td>-0.1307804</td><td>-0.0471586</td><td>-0.0639698</td><td>-0.0865672</td><td>-0.1600841</td><td>late response hypomethylation</td></tr>
<tr><td>cg17162808</td><td>PIGU (-1208)</td><td>20</td><td>33266296</td><td>PIGU</td><td>TSS1500</td><td>chr20:33264666-33265193</td><td>S_Shore</td><td>NA</td><td>0</td><td>-0.001343</td><td>-0.1057843</td><td>0.06271624</td><td>-0.0262806</td><td>0.01394189</td><td>-0.1624432</td><td>oscillatory response</td></tr>
<tr><td>cg22933807</td><td>MEIS3 (-2641)</td><td>19</td><td>47925425</td><td></td><td></td><td>chr19:47925306-47925526</td><td>Island</td><td>Egr-1|ZBTB7A_</td><td>1</td><td>-0.0277073</td><td>-0.100136</td><td>-0.0243848</td><td>-0.0325833</td><td>0.04312157</td><td>-0.1625307</td><td>late response hypomethylation</td></tr>
<tr><td>cg01150270</td><td>NONE</td><td>15</td><td>95869918</td><td></td><td></td><td>chr15:95869955-95870322</td><td>N_Shore</td><td>GR</td><td>0</td><td>-0.0213534</td><td>-0.1257846</td><td>0.09878723</td><td>-0.0186762</td><td>0.03731426</td><td>-0.1644591</td><td>oscillatory response</td></tr>
<tr><td>cg20548043</td><td>DCAF13 (-302), SLC25A32 (+828)</td><td>8</td><td>104426639</td><td>DCAF13;SLC25A32</td><td>TSS1500;Body</td><td>chr8:104426894-104427823</td><td>N_Shore</td><td>Mxi1_(bHLH)|</td><td>1</td><td>-0.033155</td><td>-0.1088668</td><td>-0.0072072</td><td>-0.0312866</td><td>0.02321813</td><td>-0.1649776</td><td>late response hypomethylation</td></tr>
<tr><td>cg23806621</td><td>BHLHE23 (-115)</td><td>20</td><td>61638501</td><td>BHLHE23</td><td>TSS200</td><td>chr20:61636171-61639055</td><td>Island</td><td>NA</td><td>0</td><td>-0.1840408</td><td>-0.1859071</td><td>-0.177053</td><td>-0.1232239</td><td>-0.2505889</td><td>-0.1667438</td><td>constant hypomethylation</td></tr>
<tr><td>cg21691166</td><td>SIGLEC15 (-49863), SLC14A1 (+51590)</td><td>18</td><td>43355681</td><td></td><td></td><td>chr18:43355318-43355698</td><td>Island</td><td>Pol2</td><td>0</td><td>0.00206315</td><td>-0.1113422</td><td>0.05218834</td><td>0.00971881</td><td>0.04126887</td><td>-0.1689115</td><td>oscillatory response</td></tr>
<tr><td>cg08194009</td><td>KSR1 (-91607), WSB1 (+86323)</td><td>17</td><td>25707428</td><td></td><td></td><td></td><td></td><td>ELF1_(SC-631)</td><td>1</td><td>-0.1446632</td><td>-0.1364068</td><td>-0.0394462</td><td>-0.1471775</td><td>-0.1123327</td><td>-0.1691295</td><td>late response hypomethylation</td></tr>
<tr><td>cg17676428</td><td>TOLLIP (+16849), MUC5B (+69748)</td><td>11</td><td>1314042</td><td>TOLLIP</td><td>Body</td><td>chr11:1315467-1317115</td><td>N_Shore</td><td>NA</td><td>0</td><td>-0.2317528</td><td>-0.1114635</td><td>-0.083926</td><td>-0.1440593</td><td>-0.1765289</td><td>-0.169519</td><td>constant hypomethylation</td></tr>
<tr><td>cg00445824</td><td>ISYNA1 (+181)</td><td>19</td><td>18548929</td><td>ISYNA1;ISYNA1;ISYNA1</td><td>5'UTR;TSS200;1stExon</td><td>chr19:18543828-18549161</td><td>Island</td><td>Pol2|TAF1|Pol</td><td>1</td><td>-0.1491034</td><td>-0.1143979</td><td>-0.0971925</td><td>-0.0834929</td><td>-0.1341804</td><td>-0.1723149</td><td>constant hypomethylation</td></tr>
<tr><td>cg10590628</td><td>PLXNA4 (-227892), CHCHD3 (+277613)</td><td>7</td><td>132489214</td><td>CHCHD3</td><td>Body</td><td></td><td></td><td>NA</td><td>0</td><td>-0.3462574</td><td>-0.3299843</td><td>-0.090181</td><td>-0.0663772</td><td>-0.1358421</td><td>-0.1740031</td><td>constant hypomethylation</td></tr>
<tr><td>cg05760722</td><td>FIGNL2 (+110462), SCN8A (+130219)</td><td>12</td><td>52115238</td><td>SCN8A</td><td>Body</td><td>chr12:52115410-52115679</td><td>N_Shore</td><td>NA</td><td>0</td><td>0.00239316</td><td>-0.1196222</td><td>0.02568562</td><td>-0.0393951</td><td>0.07590718</td><td>-0.1742023</td><td>oscillatory response</td></tr>
</table>

cg14647944	KLF9 (-66168), TRPM3 (+640773)	9	73095740					HA-E2F1|GATA	1	-0.2373601	-0.1796263	-0.0804474	-0.078376	-0.1526455	-0.1767937	constant hypomethylation
cg02082273	AMPD3 (+815)	11	10473038	AMPD3	Body	chr11:10472000-10472857	S_Shore	EBF	1	-0.0046421	-0.1742498	0.02852643	0.0544419	-0.0160888	-0.1782503	oscillatory response
cg04316537	IL1R2 (-18416), MAP4K4 (+275725)	2	102589889					NA	1	-0.0051759	-0.1202412	-0.0681103	0.00034417	0.01026652	-0.1922816	oscillatory response
cg26400835	RIC8B (-64509), RFX4 (+108975)	12	107103889	RFX4;RFX4;RFX4	Body;Body;Body			NA	0	-0.0290011	-0.1020851	0.00577254	0.03572943	-0.0855781	-0.2305351	late response hypomethylation
cg26561082	ZMYND11 (+184430), DIP2C (+325244)	10	410363	DIP2C	Body	chr10:409201-409523	S_Shore	NA	0	-0.1810535	-0.177243	-0.0406948	-0.1549523	-0.234016	-0.2310612	late response hypomethylation
cg19192256	PTGDR2 (+1729), CCDC86 (+12286)	11	60621714	GPR44	5'UTR	chr11:60619924-60621111	S_Shore	eGFP-GATA2|(	0	-0.2911395	-0.2971841	-0.1309176	-0.1277124	-0.2426533	-0.2388153	constant hypomethylation
cg06597095	CCL28 (-67874), C5orf28 (+3630)	5	43480361	C5orf28	5'UTR	chr5:43483519-43484555	N_Shelf	NA	0	-0.2725851	-0.2259461	-0.0815063	-0.1270944	-0.2314245	-0.2418151	constant hypomethylation
cg17164345	PAK1 (-86207), AQP11 (-29365)	11	77271314					BAF155|STAT3	1	-0.3426322	-0.1482034	-0.0575908	-0.1252396	-0.1919325	-0.2498506	constant hypomethylation
cg14069601	EFCAB9 (+76)	5	171621251	EFCAB9	1stExon			NA	0	-0.0996964	-0.1528196	0.13593404	-0.0203767	-0.0122552	-0.2794071	oscillatory response
cg08447739	HTRA1 (-681)	10	124220359	HTRA1	TSS1500	chr10:124220338-124222240	Island	NA	1	-0.2159981	-0.2676722	-0.0368496	-0.1822048	-0.3418058	-0.2887986	late response hypomethylation
cg20104640	ABRA (+106179), OXR1 (+216141)	8	107676292	OXR1;OXR1	Body;Body			NA	0	-0.2512685	-0.2648285	-0.1925902	-0.2051379	-0.2604451	-0.3014269	constant hypomethylation
cg18424634	ZIC1 (-83)	3	147127097	ZIC1	TSS200	chr3:147126988-147128999	Island	SUZ12|NANOG	1	-0.293494	-0.2552738	-0.0719918	-0.1498317	-0.2242581	-0.3079165	constant hypomethylation
cg17983217	DDAH2 (-185)	6	31698223	DDAH2	TSS200	chr6:31695894-31698245	Island	Pol2-4H8|Pol2	1	-0.3192856	-0.2173157	-0.203754	-0.1814438	-0.29932	-0.3218273	constant hypomethylation
cg15706807	FAM110B (-800514), IMPAD1 (-200169)	8	58106598					NA	0	-0.3161287	-0.2864641	-0.2903612	-0.2428694	-0.286256	-0.3285706	constant hypomethylation
cg04138591	FOXK1 (-373947)	7	4347982			chr7:4347707-4348017	Island	NA	0	-0.3024692	-0.2268826	-0.1835983	-0.1444321	-0.2971245	-0.3437743	constant hypomethylation
cg00984474	FASTKD3 (+18227), ADCY2 (+454580)	5	7850922	C5orf49	Body	chr5:7850957-7851413	N_Shore	NA	1	-0.4977016	-0.3669299	-0.484895	-0.3624148	-0.4724401	-0.4898058	constant hypomethylation

[illegible] Genome Res 25: 1801-1811

Supplementary table S2. iDMPs by genomic feature

	Total probes	Significant probes		Enrichment (95% CI)	P-value
All probes	425496	388	425108		
CpG island feature					
Island	136859	104	136755	0.83 (0.66 - 1.04)	1.03E-01
Shore	100342	124	100218	1.36 (1.10 - 1.66)	4.05E-03
Shelf	39420	35	39385	0.97 (0.67 - 1.38)	1.00E+00
Not island/shore/shelf	148875	125	148750	0.92 (0.75 - 1.13)	4.50E-01
Gene feature					
TSS1500	75235	84	75151	1.22 (0.96 - 1.55)	9.38E-02
TSS200	57412	41	57371	0.78 (0.55 - 1.08)	1.56E-01
5' UTR	125218	125	125093	1.09 (0.89 - 1.34)	3.71E-01
1st Exon	35161	107	35054	3.34 (2.67 - 4.15)	1.11E-22
Gene body	156844	125	156719	0.87 (0.71 - 1.07)	1.96E-01
3' UTR	17539	14	17525	0.88 (0.47 - 1.49)	7.97E-01
DNase I hypersensitivity sites	199995	173	199822	0.95 (0.79 - 1.14)	5.87E-01
Transcription factor binding sites	203540	171	203369	0.92 (0.76 - 1.11)	3.90E-01

Supplementary Table 3. Comparison with publically available DNA methylation changes during infection with M. tuberculosis. Genes annotated to the iDMPs in *B. pseudomallei* and in *M. tuberculosis* infections

**Genes annotated to the iDMPs in *B. pseudomallei* and in *M. tuberculosis* infections**		**Differential methylation pattern in *B. pseudomallei* infection**					**Differential methylation pattern in *M. tuberculosis* infection[1]**						
**Hugo Gene ID**	**Ensembl Gene ID**	**Chromosome**	**Start**	**End**	**Distance from TSS**	**Direction**	**Chromosome**	**Start**	**End**	**# of CpGs**	**Length bp**	**Distance from TSS**	**Direction**
ABRA	ENSG00000174429	chr8	107676291	107676292	(+106179)	constant hypomethylation	chr8	107823512	107823795	4	284	41179	hypo
IMPAD1	ENSG00000104331	chr8	58106597	58106598	(-200169)	constant hypomethylation	chr8	58255707	58255727	3	21	349313	hypo
IRF8	ENSG00000140968	chr16	85966236	85966237	(+33464)	constant hypomethylation	chr16	86017636	86017715	4	80	85266	hyper
GMDS	ENSG00000112699	chr6	1616108	1616109	(+629758)	early response hypermethylation	chr6	1654600	1654713	3	114	30615	hypo
							chr6	2286018	2286209	3	192	40186	hypo
							chr6	2375802	2375895	7	94	129921	hyper
PCDHGC5	ENSG00000240764	chr5	140773538	140773539	(-95268)	early response hypermethylation	chr5	140874572	140874742	7	171	5849	hyper
PPP1R14C	ENSG00000198729	chr6	150547953	150547954	(+83767)	early response hypermethylation	chr6	150489390	150489400	4	11	25183	hypo
PTH1R	ENSG00000160801	chr3	46933807	46933808	(+10070)	early response hypermethylation	chr3	46940550	46940663	5	114	21370	hypo
ZFHX3	ENSG00000140836	chr16	73098173	73098174	(-15901)	early response hypermethylation	chr16	72968586	72968803	8	218	151910	hyper
							chr16	73045820	73045944	3	125	229098	hyper
PAX3	ENSG00000135903	chr2	223177573	223177574	(-13860)	early response hypomethylation	chr2	223196957	223197049	4	93	33287	hypo
PCTP	ENSG00000141179	chr17	53810812	53810813	(-17526)	early response hypomethylation	chr17	53923046	53923053	4	8	94709	hyper
SPOCK2	ENSG00000107742	chr10	73899797	73899798	(-51268)	early response hypomethylation	chr10	73808050	73808087	4	38	40721	hypo
							chr10	73793980	73794287	9	308	54656	hypo
CDHR2	ENSG00000074276	chr5	175975900	175975901	(-441)	late response hypermethylation	chr5	175997165	175997305	3	141	27723	hyper
KCNH5	ENSG00000140015	chr14	62584004	62584005	(+927949)	late response hypermethylation	chr14	63178366	63178634	3	269	390254	hypo
SYT16	ENSG00000139973	chr14	62584004	62584005	(+121465)	late response hypermethylation	chr14	62361376	62361531	3	156	92348	hypo
CDC42BPA	ENSG00000143776	chr1	227163995	227163996	(+341829)	late response hypomethylation	chr1	227447176	227447302	6	127	269673	hypo
CDKN1C	ENSG00000129757	chr11	2890724	2890725	(+16269)	late response hypomethylation	chr11	2872680	2872786	3	107	34377	hypo
							chr11	2688467	2688525	4	59	218614	hypo
FOXP1	ENSG00000114861	chr3	70360571	70360572	(+933743)	late response hypomethylation	chr3	71029827	71030284	6	458	26211	hypo
							chr3	71388273	71388360	3	88	384472	hyper
GABRP	ENSG00000094755	chr5	169929559	169929560	(-281162)	late response hypomethylation	chr5	170171941	170171956	3	16	18404	hypo
KCNQ1	ENSG00000053918	chr11	2890724	2890725	(+424505)	late response hypomethylation	chr11	2497906	2498009	3	104	32043	hyper
KCTD17	ENSG00000100379	chr22	37448204	37448205	(+427)	late response hypomethylation	chr22	37436891	37436981	3	91	10841	hyper
KSR1	ENSG00000141068	chr17	25707427	25707428	(-91607)	late response hypomethylation	chr17	25867805	25868018	5	214	84241	hyper
LRRC4C	ENSG00000148948	chr11	40137954	40137955	(+177708)	late response hypomethylation	chr11	40717157	40717351	3	195	581411	hyper
							chr11	39354476	39354607	3	132	2126781	hypo
MANSC1	ENSG00000111261	chr12	12518874	12518875	(-15707)	late response hypomethylation	chr12	12486910	12487145	3	236	4829	hypo
MR1	ENSG00000153029	chr1	181128763	181128764	(+126204)	late response hypomethylation	chr1	181012584	181012797	3	214	9623	hypo
NCOR2	ENSG00000196498	chr12	125138262	125138263	(-86254)	late response hypomethylation	chr12	125094113	125094228	4	116	42159	hypo
							chr12	125101198	125101264	3	67	49220	hyper
							chr12	125137761	125137796	5	36	85767	hypo
							chr12	124959931	124960125	4	195	128889	hypo
							chr12	125030049	125030131	5	83	198951	hyper
							chr12	124817913	124817943	3	31	234081	hyper
PARD6G	ENSG00000178184	chr18	77905407	77905408	(+99988)	late response hypomethylation	chr18	78015897	78016160	4	264	10598	hyper
PEX26	ENSG00000215193	chr22	18531446	18531447	(-29238)	late response hypomethylation	chr22	18586766	18587005	5	240	26196	hypo
RAC1	ENSG00000136238	chr7	6460965	6460966	(+46841)	late response hypomethylation	chr7	6422909	6423020	3	112	8810	hypo
							chr7	6427547	6427646	3	100	13442	hypo
SCARB1	ENSG00000073060	chr12	125138262	125138263	(+210255)	late response hypomethylation	chr12	125251184	125251208	3	25	97266	hypo
SSTR5	ENSG00000162009	chr16	1116360	1116361	(-6394)	late response hypomethylation	chr16	1080397	1080432	4	36	42340	hypo
TNS3	ENSG00000136205	chr7	47780843	47780844	(-159103)	late response hypomethylation	chr7	47493861	47493996	4	136	151363	hyper
							chr7	47583350	47583560	6	211	240890	hypo
ACTR3	ENSG00000115091	chr2	114644206	114644207	(-3329)	oscillatory response	chr2	114769434	114769576	3	143	121968	hyper
AGAP1	ENSG00000157985	chr2	236839266	236839267	(+436535)	oscillatory response	chr2	236208765	236208773	3	9	193962	hyper
ALPK2	ENSG00000198796	chr18	56067461	56067462	(+228726)	oscillatory response	chr18	56217161	56217219	4	59	68711	hyper

AMPD3	ENSG00000133805	chr11	10473037	10473038	(+815)	oscillatory response	chr11	10336499	10336519	3	21	2707	hyper
							chr11	10383917	10384302	3	386	44891	hyper
ANKMY1	ENSG00000144504	chr2	241392616	241392617	(+104787)	oscillatory response	chr2	241504215	241504290	3	76	32759	hypo
ANO5	ENSG00000171714	chr11	22213802	22213803	(-918)	oscillatory response	chr11	22191262	22191541	3	280	23319	hypo
AUH	ENSG00000148090	chr9	93588841	93588842	(+535363)	oscillatory response	chr9	93929129	93929351	4	223	194954	hyper
BMPR1B	ENSG00000138696	chr4	96465501	96465502	(+786375)	oscillatory response	chr4	96060847	96060891	6	45	381750	hyper
C1QTNF1	ENSG00000173918	chr17	77029307	77029308	(+9058)	oscillatory response	chr17	77034940	77035170	5	231	16159	hypo
CCR4	ENSG00000183813	chr3	32864306	32864307	(-128758)	oscillatory response	chr3	33034083	33034475	7	393	41213	hypo
COG6	ENSG00000133103	chr13	41070899	41070900	(+841137)	oscillatory response	chr13	40600338	40600585	5	248	370697	hypo
CYTH1	ENSG00000108669	chr17	76672155	76672156	(+106219)	oscillatory response	chr17	76730931	76731264	5	334	60967	hypo
DIO3	ENSG00000197406	chr14	101520661	101520662	(-507025)	oscillatory response	chr14	101908984	101909082	8	99	118653	hypo
DKK2	ENSG00000155011	chr4	107956954	107956955	(+497)	oscillatory response	chr4	107955060	107955076	3	17	109966	hypo
DMRT3	ENSG00000064218	chr9	995578	995579	(+18616)	oscillatory response	chr9	969337	969350	3	14	7619	hyper
DNAH17	ENSG00000187775	chr17	76672155	76672156	(-98681)	oscillatory response	chr17	76439374	76439426	4	53	19622	hypo
DNAJB6	ENSG00000105993	chr7	157658871	157658872	(+529163)	oscillatory response	chr7	157103424	157103654	3	231	24534	hypo
							chr7	157089794	157089894	3	101	38229	hypo
							chr7	157188998	157189053	5	56	60950	hyper
DOCK10	ENSG00000135905	chr2	225907605	225907606	(-277)	oscillatory response	chr2	225902739	225903147	5	409	144678	hypo
DTNBP1	ENSG00000047579	chr6	15365073	15365074	(+298214)	oscillatory response	chr6	15549755	15549833	4	79	26720	hypo
EHHADH	ENSG00000113790	chr3	184870892	184870893	(+100992)	oscillatory response	chr3	184934163	184934268	3	106	25803	hypo
ESRRB	ENSG00000119715	chr14	76617754	76617755	(-219934)	oscillatory response	chr14	76734564	76734651	5	88	42348	hyper
							chr14	76948653	76948822	3	170	171780	hyper
EZH2	ENSG00000106462	chr7	148537902	148537903	(+43537)	oscillatory response	chr7	148579004	148579262	3	259	64004	hyper
FOXO1	ENSG00000150907	chr13	41070899	41070900	(+169833)	oscillatory response	chr13	41148152	41148449	4	298	18496	hypo
							chr13	41193536	41193626	4	91	63777	hypo
GNPDA1	ENSG00000113552	chr5	141392794	141392795	(-176)	oscillatory response	chr5	141408154	141408394	6	241	15667	hyper
IGF1R	ENSG00000140443	chr15	99385322	99385323	(+192563)	oscillatory response	chr15	99446590	99446756	6	167	254473	hypo
IRX4	ENSG00000113430	chr5	1876167	1876168	(+6711)	oscillatory response	chr5	1912261	1912363	3	103	24961	hypo
							chr5	1924636	1924841	5	206	37387	hyper
JARID2	ENSG00000008083	chr6	15365073	15365074	(+118548)	oscillatory response	chr6	15167607	15167965	3	359	78739	hypo
							chr6	14938771	14939209	4	439	307535	hyper
							chr6	14875764	14876085	6	322	370601	hypo
KLF14	ENSG00000174595	chr7	130600074	130600075	(-181188)	oscillatory response	chr7	130572190	130572336	6	147	153374	hypo
LIN28B	ENSG00000187772	chr6	105391856	105391857	(-13065)	oscillatory response	chr6	105421367	105421520	3	154	16520	hyper
LOR	ENSG00000203782	chr1	153234267	153234268	(+2090)	oscillatory response	chr1	153235491	153235736	3	246	3437	hypo
MAP4K4	ENSG00000071054	chr2	102589888	102589889	(+275725)	oscillatory response	chr2	102364910	102365077	4	168	51681	hyper
							chr2	102254700	102254767	3	68	58577	hypo
							chr2	102246790	102247002	8	213	66414	hypo
MKLN1	ENSG00000128585	chr7	130600074	130600075	(-412519)	oscillatory response	chr7	130716302	130716734	8	433	78335	hypo
							chr7	130878860	130879328	4	469	84239	hyper
MTRF1	ENSG00000120662	chr13	41837662	41837663	(+49)	oscillatory response	chr13	41837188	41837284	3	97	46022	hyper
NEDD4L	ENSG00000049759	chr18	56067461	56067462	(+355853)	oscillatory response	chr18	55988849	55988875	3	27	277263	hypo
OR2V2	ENSG00000182613	chr5	180597641	180597642	(+15700)	oscillatory response	chr5	180601738	180601754	3	17	19803	hyper
PAWR	ENSG00000177425	chr12	79716681	79716682	(+368107)	oscillatory response	chr12	79959674	79959730	6	57	125174	hypo
PGPEP1L	ENSG00000183571	chr15	99385322	99385323	(+163561)	oscillatory response	chr15	99552368	99552384	3	17	1351	hyper
PLEKHG4B	ENSG00000153404	chr5	192649	192650	(+52278)	oscillatory response	chr5	12466	12483	6	18	127897	hypo
PMP22	ENSG00000109099	chr17	15164269	15164270	(-178)	oscillatory response	chr17	15174542	15174725	3	184	5989	hypo
							chr17	15165657	15165670	4	14	31279	hypo
PRKCH	ENSG00000027075	chr14	61790036	61790037	(+1523)	oscillatory response	chr14	61657312	61657492	6	181	3125	hypo
							chr14	61578052	61578144	11	93	76177	hypo
PRKCZ	ENSG00000067606	chr1	2125975	2125976	(+144068)	oscillatory response	chr1	2077884	2077967	3	84	96016	hyper
PTDSS1	ENSG00000156471	chr8	97270148	97270149	(-4017)	oscillatory response	chr8	97343908	97344017	4	110	70019	hypo
PTPRN2	ENSG00000155093	chr7	157658871	157658872	(+721609)	oscillatory response	chr7	157550616	157550679	3	64	218897	hyper

RFXAP	ENSG00000133111	chr13	37269128	37269129	(-124209)	oscillatory response	chr13	37394390	37394639	4	250	1153	hypo
RUFY1	ENSG00000176783	chr5	179002267	179002268	(+24698)	oscillatory response	chr5	178980927	178980992	3	66	3400	hyper
SEC61G	ENSG00000132432	chr7	54615863	54615864	(+211074)	oscillatory response	chr7	54856843	54857043	3	201	29275	hypo
SLC4A8	ENSG00000050438	chr12	51818680	51818681	(+88)	oscillatory response	chr12	51792460	51792690	8	231	7474	hypo
SMAP1	ENSG00000112305	chr6	71605995	71605996	(+228518)	oscillatory response	chr6	71376516	71376649	5	134	895	hyper
SORCS2	ENSG00000184985	chr4	7301175	7301176	(+106803)	oscillatory response	chr4	7298967	7299134	6	168	104785	hyper
							chr4	7628337	7628455	4	119	434131	hypo
							chr4	7697748	7698182	4	435	503700	hyper
SOX4	ENSG00000124766	chr6	21770732	21770733	(+176762)	oscillatory response	chr6	21438636	21438667	3	32	155319	hypo
							chr6	21402797	21402890	4	94	191127	hyper
							chr6	21377963	21378176	5	214	215901	hypo
SPATA19	ENSG00000166118	chr11	133800240	133800241	(-84850)	oscillatory response	chr11	133617339	133617453	3	115	98036	hypo
SPATS2L	ENSG00000196141	chr2	201171254	201171255	(-100)	oscillatory response	chr2	201202770	201202973	3	204	32267	hypo
SYK	ENSG00000165025	chr9	93588841	93588842	(+24831)	oscillatory response	chr9	93818861	93819105	4	245	254914	hypo
TBX3	ENSG00000135111	chr12	115134279	115134280	(-12312)	oscillatory response	chr12	115129242	115129287	3	46	7294	hyper
TEAD1	ENSG00000187079	chr11	12695903	12695904	(-64)	oscillatory response	chr11	12916161	12916210	3	50	220216	hypo
TRIM27	ENSG00000204713	chr6	28584463	28584464	(+307303)	oscillatory response	chr6	28885706	28885898	3	193	15023	hyper
							chr6	28863663	28863725	6	63	28071	hypo
UNC5C	ENSG00000182168	chr4	96465501	96465502	(+4858)	oscillatory response	chr4	96133793	96133847	3	55	50165	hyper
UPK3B	ENSG00000243566	chr7	76145671	76145672	(+5928)	oscillatory response	chr7	76608553	76608591	4	39	468827	hypo
VENTX	ENSG00000151650	chr10	135049207	135049208	(-2199)	oscillatory response	chr10	135066730	135066839	3	110	15876	hyper
VRK1	ENSG00000100749	chr14	97267125	97267126	(+3443)	oscillatory response	chr14	97527858	97527936	3	79	264256	hyper
							chr14	97684742	97684839	4	98	421149	hyper
YWHAE	ENSG00000108953	chr17	1214950	1214951	(+88604)	oscillatory response	chr17	1258300	1258349	3	50	10758	hypo
ZFP42	ENSG00000179059	chr4	188427965	188427966	(-488958)	oscillatory response	chr4	188954042	188954058	4	17	37125	hypo
APBB1IP	ENSG00000077420	chr10	26856453	26856454	(+129189)	transient response hypermethylation	chr10	26791915	26792292	6	378	64971	hyper
COL5A1	ENSG00000130635	chr9	137624092	137624093	(+90442)	transient response hypermethylation	chr9	137657888	137658030	3	143	124339	hyper
DUSP4	ENSG00000120875	chr8	29206516	29206517	(+1749)	transient response hypermethylation	chr8	29240789	29241096	4	308	32756	hyper
							chr8	29294558	29294648	3	91	86417	hyper
							chr8	29357593	29357818	7	226	149519	hypo
							chr8	29381283	29381602	6	320	173256	hypo
KEAP1	ENSG00000079999	chr19	10572361	10572362	(+41691)	transient response hypermethylation	chr19	10616221	10616236	4	16	1810	hyper
KIF13B	ENSG00000197892	chr8	29206516	29206517	(-85908)	transient response hypermethylation	chr8	29028288	29028332	4	45	24935	hypo
							chr8	28970095	28970313	3	219	150436	hypo
LAMA1	ENSG00000101680	chr18	6929789	6929790	(+188022)	transient response hypermethylation	chr18	7138503	7138519	3	17	20697	hyper
MDM1	ENSG00000111554	chr12	68729044	68729045	(-2885)	transient response hypermethylation	chr12	68763779	68763987	4	209	37721	hypo
MEF2C	ENSG00000081189	chr5	87985588	87985589	(+214332)	transient response hypermethylation	chr5	88028895	88029125	3	231	14971	hyper
NTRK3	ENSG00000140538	chr15	88420437	88420438	(+379523)	transient response hypermethylation	chr15	88580558	88580724	3	167	162411	hypo
PDSS1	ENSG00000148459	chr10	26856453	26856454	(-130140)	transient response hypermethylation	chr10	27025927	27026218	3	292	39484	hypo
							chr10	27029189	27029488	3	300	42750	hypo
QRFPR	ENSG00000186867	chr4	122137695	122137696	(+164484)	transient response hypermethylation	chr4	122282828	122283020	5	193	32455	hypo
CELF2	ENSG00000048740	chr10	11317786	11317786	(+257895)	transient response	chr10	11241680	11242283	8	604	194722	hypo
		chr10		11504218	(+444327)	hypermethylation	chr10	11318184	11318867	14	684	271266	hypo
						oscillatory response							
RBFOX3	ENSG00000167281	chr17	77644544	77644545	(-165866)	transient response	chr17	77389312	77389320	3	9	302418	hypo
		chr17	77078090	77078091	(+400588)	hypermethylation							
						oscillatory response							
USP6NL	ENSG00000148429	chr10	11317786	11317787	(+256486)	transient response	chr10	11625479	11625485	3	7	129537	hyper
		chr10	11504218	11504219	(+70054)	hypermethylation	chr10	11423659	11423683	3	25	230081	hypo
						oscillatory response	chr10	11386849	11386921	7	73	266867	hypo
CRADD	ENSG00000169372	chr12	94244495	94244496	(+173346)	transient response hypomethylation	chr12	94018722	94019073	3	352	52252	hypo
							chr12	94179109	94179216	4	108	108011	hypo
DDX39A	ENSG00000123136	chr19	14543882	14543883	(-13689)	transient response hypomethylation	chr19	14532973	14533238	3	266	2925	hypo

FERMT2	ENSG00000073712	chr14	53421043	53421044	(-3230)	transient response hypomethylation	chr14	53474193	53474327	3	135	55106	hypo
IRX5	ENSG00000176842	chr16	54352340	54352341	(-612769)	transient response hypomethylation	chr16	55090476	55090512	3	37	125720	hyper
LDB2	ENSG00000169744	chr4	16847843	16847844	(+52579)	transient response hypomethylation	chr4	16900733	16900813	3	81	340	hypo
LRAT	ENSG00000121207	chr4	155661848	155661849	(-3313)	transient response hypomethylation	chr4	155598911	155599008	3	98	50846	hyper
							chr4	155599340	155599397	3	58	51255	hyper
NKX6-2	ENSG00000148826	chr10	134738297	134738298	(-138762)	transient response hypomethylation	chr10	134649562	134649602	3	41	50025	hyper
PLXNC1	ENSG00000136040	chr12	94244495	94244496	(-298002)	transient response hypomethylation	chr12	94602291	94602316	4	26	59804	hypo
							chr12	94656787	94656885	6	99	114337	hypo
PPM1L	ENSG00000163590	chr3	160569627	160569628	(+95633)	transient response hypomethylation	chr3	160518851	160519105	3	255	45588	hypo
SAMD4A	ENSG00000020577	chr14	55034645	55034646	(+317)	transient response hypomethylation	chr14	55036376	55036470	3	95	2608	hyper
TRIM2	ENSG00000109654	chr4	154075470	154075471	(-50126)	transient response hypomethylation	chr4	154028504	154028728	3	225	45027	hyper
TTC34	ENSG00000215912	chr1	2904594	2904595	(-198366)	transient response hypomethylation	chr1	2722280	2722321	3	42	4013	hypo
							chr1	2780762	2780836	4	75	62512	hyper
BCL11B	ENSG00000127152	chr14	99738492	99738493	(-672)	transient response hypomethylation	chr14	99541066	99541100	3	35	196777	hyper
		chr14	99766300	99766301	(-28480)	oscillatory response							
DBX1	ENSG00000109851	chr11	20182694	20182695	(-826)	transient response hypomethylation	chr11	20149888	20149961	3	74	32234	hypo
		chr11	20182121	20182122	(-253)	transient response hypomethylation							

[1] Pacis A, Tailleux L, Morin AM, Lambourne J, MacIsaac JL, et al. (2015) Bacterial infection remodels the DNA methylation landscape of human dendritic cells. Genome Res 25: 1801–1811.

Supplementary Table 4. Comparison with publically available transciption data. Genes annotated to conserved iDMPs in our study and differentially expressed in patients with septisemic melioidosis[1]

					The expression profiles of genes, which were modulated in the whole blood of patients with septicemic melioidosis (Pankla *et al.*) [1]	
**Gene annotation using GREAT**	**Chromosome**	**Location**	**Distance from TSS**	**Differential methylation pattern**	**Log fold-change**	**Fold-change p-value**
ABCE1	4	145568306	(-450849),	transient response hypomethylation	-1.8	0.000968
ADCK3	1	227163996	(+36059),	late response hypomethylation	-1.3	0.000968
ADRB2	5	148034206	(-171949),	oscillatory response	-1.4	0.00139
AMPD3	11	10473038	(+815)	oscillatory response	1.1	0.00495
ANKMY1	2	241392617	(+104787)	oscillatory response	-1.2	0.00284
BCL11B	14	99766301	(-28480),	oscillatory response	-2.7	0.000968
BCL11B	14	99738493	(-672)	transient response hypomethylation	-2.7	0.000968
C5orf28	5	43480361	(+3630)	constant hypomethylation	-1.3	0.00139
CACNA1E	1	181128764	(-323921)	late response hypomethylation	3.3	0.000968
CACNA1E	1	181767609	(+314924)	early response hypomethylation	3.3	0.000968
CD44	11	35158283	(-2133)	oscillatory response	0.9	0.0072
CDKN1C	11	2890725	(+16269),	late response hypomethylation	-1.9	0.00284
CHSY1	15	101593341	(+198795)	oscillatory response	0.9	0.00411
CIT	12	120315669	(-575)	oscillatory response	1.1	0.00495
DCAF13	8	104426639	(-302),	late response hypomethylation	-1.1	0.00869
DDAH2	6	31698223	(-185)	constant hypomethylation	2.4	0.000968
DNAJC19	3	181182210	(-474649),	transient response hypermethylation	-1.3	0.000968
DOCK10	2	225907606	(-277)	oscillatory response	-1.4	0.000968
DTNBP1	6	15365074	(+298214)	oscillatory response	1.1	0.00342
E2F6	2	11623286	(-16990)	oscillatory response	-1.1	0.00284
ELF2	4	140060774	(-124)	oscillatory response	-1.6	0.000968
FBN2	5	127909589	(-35855)	transient response hypomethylation	1.3	0.00597
FBXO31	16	86715554	(+701839)	late response hypomethylation	-1.5	0.000968
FOXC1	6	1616109	(+5429),	early response hypermethylation	1.7	0.0012
GABPB2	1	151040405	(-2674)	oscillatory response	-1.3	0.000968
GLMN	1	92947035	(-182470),	transient response hypermethylation	-1.5	0.000968
H2AFJ	12	14928083	(+814)	oscillatory response	1.8	0.00104
HDAC4	2	239847497	(+475145)	oscillatory response	1.4	0.000968
HIF1A	14	61790037	(-372081)	oscillatory response	1.1	0.00342
HLX	1	221509763	(+457021)	transient response hypomethylation	1.2	0.00139
HNRNPH1	5	179002268	(+48453)	oscillatory response	-0.9	0.00104
IER3	6	30761828	(-49502)	oscillatory response	1.8	0.00411
IL18RAP	2	103039542	(+4289)	oscillatory response	2.7	0.000968

IL1R2	2	102589889	(-18416),	oscillatory response	3.5	0.00104
IRF8	16	85966237	(+33464)	constant hypomethylation	-1	0.00411
KCTD17	22	37448205	(+427)	late response hypomethylation	-1.5	0.00163
MAEA	4	1336407	(+52736)	transient response hypomethylation	0.8	0.00597
MAF	16	79632784	(+1837)	transient response hypomethylation	-1.8	0.0012
MANSC1	12	12518875	(-15707),	late response hypomethylation	1.6	0.00284
MARCH1	4	165304473	(-67)	oscillatory response	1.5	0.000968
MEF2C	5	87985589	(+214332)	transient response hypermethylation	-1.7	0.000968
METTL7B	12	56075089	(-240)	early response hypomethylation	2.9	0.00163
MIS18A	21	33248032	(+403343)	oscillatory response	-1.2	0.0012
MR1 (+126204)	1	181128764	(+126204)	late response hypomethylation	1.3	0.00163
MSRA	8	9760427	(-151402),	transient response hypermethylation	1.1	0.0012
MTAP	9	21559551	(-243083),	late response hypermethylation	-1.7	0.00104
MTERFD1	8	97270149	(+3646)	oscillatory response	-1.3	0.000968
NBEAL2	3	46933808	(-87364),	early response hypermethylation	1.4	0.000968
NMUR1	2	232395269	(-88)	oscillatory response	-1.4	0.00104
NOV	8	120429518	(+967)	transient response hypomethylation	-2.8	0.00342
NT5E	6	85824196	(-335105)	late response hypomethylation	-1.7	0.000968
PASK	2	242051808	(+37110),	oscillatory response	-2.4	0.000968
PFKFB2	1	207226830	(+211)	oscillatory response	4.9	0.000968
PKD2	4	88929658	(+860)	oscillatory response	-1	0.00495
PLXNC1	12	94244496	(-298002),	transient response hypomethylation	1.3	0.000968
PMP22	17	15164270	(-178)	oscillatory response	-1.9	0.000968
PNPLA6	19	7598793	(-797)	oscillatory response	1	0.00495
PRKCH	14	61790037	(+1523)	oscillatory response	-1.9	0.000968
PTCH1	9	98271957	(-1127)	oscillatory response	-1.7	0.00195
PTGDR2	11	60621714	(+1729),	constant hypomethylation	-2.2	0.00163
PVRL3	3	110789788	(-817)	transient response hypomethylation	-1.1	0.00139
RFXAP	13	37269129	(-124209),	oscillatory response	-1.1	0.00104
SCARB1	12	125138263	(+210255)	late response hypomethylation	-1.3	0.00411
SLC22A17	14	23821902	(-243)	transient response hypomethylation	3.4	0.000968
SLC4A8	12	51818681	(+88)	oscillatory response	-1.1	0.00139
SMAGP	12	51664655	(-454)	early response hypermethylation	-1	0.00869
SPOCK2	10	73899798	(-51268)	early response hypomethylation	-2.3	0.000968
STK3	8	99951420	(-113512),	oscillatory response	1.8	0.000968
TNFSF8	9	117657237	(+35637)	late response hypomethylation	-1.4	0.0072
TOLLIP	11	1314042	(+16849),	constant hypomethylation	1.1	0.00139
TRIM27	6	28584464	(+307303)	oscillatory response	0.9	0.00139
VENTX	10	135049208	(-2199)	oscillatory response	-1.1	0.0072
WSB1	17	25707428	(+86323)	late response hypomethylation	1.8	0.00342
ZDHHC11	5	851101	(-1)	early response hypermethylation	-1.2	0.00495
ZFHX3	16	73098174	(-15901)	early response hypermethylation	-1.3	0.00495

ZMYND11	10	237794	(+11861),	oscillatory response	-1.8	0.0012
ZMYND11	10	410363	(+184430),	late response hypomethylation	-1.8	0.0012
ZNF559	19	9435206	(+759)	oscillatory response	-1.4	0.000968

[1] Pankla R, Buddhisa S, Berry M, Blankenship DM, Bancroft GJ, et al. (2009) Genomic transcriptional profiling identifies a candidate blood biomarker signature for the diagnosis of septicemic melioidosis. Genome Biol 10: R127.
